# The DIPP1 family binds IP_8_ in catalytically-productive twist-boat and chair conformations and associates in a ligand-dependent manner

**DOI:** 10.1016/j.ijbiomac.2026.152715

**Published:** 2026-05-26

**Authors:** David Casas-Florez, Hayley Whitfield, Jose M. Pérez-Cañadillas, Begoña Monterroso, Andrew M. Riley, María A. Márquez-Moñino, Megan L. Shipton, Julia Sanz-Aparicio, Charles A. Brearley, Barry V.L. Potter, Beatriz González

**Affiliations:** 1 Department of Crystallography and Structural Biology, Institute of Physical-Chemistry Blas Cabrera, CSIC, Serrano 119, 28006 Madrid, Spain; 2 School of Biological Sciences, University of East Anglia, Norwich Research Park, Norwich NR4 7TJ, UK; 3 Department of Biological Physical Chemistry, Institute of Physical-Chemistry Blas Cabrera, CSIC, Serrano 119, 28006 Madrid, Spain; 4 Drug Discovery & Medicinal Chemistry, Department of Pharmacology, University of Oxford Mansfield Road, Oxford, OX1 3QT, UK

**Keywords:** Inositol polyphosphate, inositol pyrophosphate, IP_8_, DIPP1, Nudix hydrolase, twist-boat

## Abstract

Diphosphoinositol Polyphosphate Phosphohydrolase 1 (DIPP1) is a Nudix hydrolase involved in inositol pyrophosphate (PP-InsP) metabolism, critical for cellular signaling, energy homeostasis, and stress responses. We report crystallographic and computational studies that reveal 1,5-bis-diphosphoinositol tetrakisphosphate (IP_8_) binds to DIPP1 in two catalytically-productive inositol ring conformations. IP_8_ hydrolysis at the 1-position requires a twist-boat conformation, whereas at the 5-position a canonical chair conformation is adopted. Additionally, structural and biophysical characterization shows that the DIPP1 family undergoes ligand-sensitive changes in the association state that might be further modulated by salt concentration and/or phosphate ions. Taken together, these results advance our understanding of DIPP1 in the dynamic regulation of inositol pyrophosphate signaling networks. They provide a detailed view of DIPP1 substrate recognition and suggest oligomerization as a novel regulatory mechanism, with broader implications for phosphate sensing and functional protein–protein interactions.

## Introduction

DIPP1 (Diphosphoinositol Polyphosphate Phosphohydrolase 1) belongs to the Nudix hydrolase family, which comprises a diverse group of enzymes essential for cellular metabolism by hydrolyzing a wide variety of energy-rich substrates [1]. These substrates include nucleoside diphosphates linked to other moieties, polyphosphates (polyPs), and inositol pyrophosphates (PP-InsPs), the latter molecules being critical for intracellular signaling and metabolic regulation. Specifically, DIPP1 modulates the levels of inositol pyrophosphates [2,3], such as 1- or 5-diphosphoinositol pentakisphosphate (1-PP-IP_5_ or 1-IP_7_; and 5-PP-IP_5_ or 5-IP_7_) and 1,5-bis-diphosphoinositol tetrakisphosphate (1,5-[PP]_2_-IP_4_ or IP_8_), which are central players in cellular signaling and bioenergetics. In particular, these molecules are involved in phosphate homeostasis, energy metabolism, and stress responses, as well as protein pyrophosphorylation [4–8]. Dysregulation of PP-InsP metabolism has been linked to several physiological and pathological conditions, including cancer, obesity, and aging [4]. The regulatory impact of PP-InsPs on cellular pathways underscores the importance of maintaining their homeostasis, a function mediated, among others, by DIPP enzymes. DIPP1 hydrolyzes PP-InsP diphosphate bonds, converting PP-InsPs into the inositol polyphosphate (InsP) product inositol hexakisphosphate (IP_6_), and thereby influencing the intracellular concentrations of these signaling molecules and modulating downstream effects. Interestingly, this family also exhibits high catalytic efficiency toward inorganic polyPs [9,10], an ability retained by human DIPP1 (*Hs*DIPP1) and a land plant orthologue [11].

DIPP1 shares the canonical Nudix fold consisting of a mixed β-sheet core flanked by α-helices [12]. It needs divalent cations such as magnesium (Mg^2+^) for its enzymatic activity and there is evidence that other divalent cations could also influence substrate binding and catalytic efficiency [13]. Notably, this activity is inhibited by fluoride anion [12]. Zong *et al*. [14] provided substantial kinetic analysis for PP-InsP binding to *Hs*DIPP1, revealing that this enzyme preferentially hydrolyzes 1-IP_7_ over 5-IP_7_. Additionally, *Hs*DIPP1 is able to hydrolyze IP_8_ at both diphosphate positions (1 and 5, Fig. S1), in contrast to PPIP5K (diphosphoinositol pentakisphosphate kinase), which hydrolyzes IP_8_ specifically in the 1-PP moiety [15].

Studies by X-ray crystallography have revealed the molecular details of substrate recognition in DIPP1, highlighting the role of positively charged residues in the active site that stabilize the PP-InsPs polyanions [12,14]. Although we understand the basis for IP_7_ molecular recognition and productive hydrolysis for 1-PP and 5-PP bonds by *Hs*DIPP1 [14] and the yeast homologue *Sc*DDP1 [16], the molecular details of IP_8_ binding remain less understood. While a structure showing IP_8_ in a productive mode for 1-PP or 5-PP hydrolysis is not available, studies with *Sc*DDP1 revealed that a non-hydrolyzable IP_8_ analogue, PCP-IP_8_, can bind within its active site in a semi-productive mode for 1-PP hydrolysis. In this mode, PCP-IP_8_ interacts with the key enzymatic residues for substrate binding, but does not adopt an expected fit for efficient catalysis [16]. Interestingly, Kurz *et al* [17] suggested that under cytosolic conditions, IP_8_ actively interconverts between two chair conformations of its cyclitol ring (1axial/5equatorial and the flipped 1equatorial/5axial) with more than 30% of the cytosolic pool adopting the flipped conformation featuring five axial substituents. Previous studies with PCP-IP_8_[18] and with 1,3,4,5-inositol tetrakisphosphate (IP_4_) [19] also revealed such flipping events. This opens a scenario for IP_8_ conformational variability to avoid steric clashes with DIPP1.

Interestingly, though the DIPP1 family presents high active site conservation across the species, members show some important differences. For example, the yeast orthologue *Sc*DDP1 possesses unique insertions, such as the "nose" extension, proposed to define the polyphosphate binding or even protein-protein interactions based in a crystallographic head-to-tail arrangement [16]. This and other distinct features underscore the structural diversity within this enzyme family that require experimental structural data to elucidate their functional implications.

Despite the great advances to date in DIPP1 biology, some critical questions about function and regulation still remain unanswered. First, no productive binding mode for IP_8_ has been described for DIPP1, leaving a significant gap in the rationalization of substrate recognition and specificity. Secondly, the mechanisms underlying DIPP1 regulation are unknown. In this study, we address these gaps using a multidisciplinary approach with X-ray crystallography, molecular docking and dynamics, NMR, site-directed mutagenesis, enzymatic activity assays and a combination of biophysical methods such as differential scanning fluorimetry (DSF), analytical ultracentrifugation (AUC) and dynamic light scattering (DLS). Thus, we provide new insights into the molecular basis of DIPP1 substrate recognition and explore the role of DIPP1 self-association as a potential regulatory mechanism. Together, these results represent a step forward to draw a more complete picture of the role of DIPP1 in inositol pyrophosphate metabolism and, hence, to envision its broader implications in cellular physiology.

## Materials and methods

### PP-InsPs synthesis

5-IP_7_ was synthesized as previously described [20]. 1-IP_7_ and IP_8_ were synthesized in a similar way, using the appropriate chiral penta-*O*-benzyl and tetra-*O*-benzyl *myo*-inositol precursors, respectively. PCP-IP_8_ was synthesized as previously described [21] and PCP-IP_7_ (= 1-PCP-IP_5_) was synthesized similarly [16]. These compounds were purified by ion-exchange chromatography and characterized by ^1^H, ^31^P, and ^13^C nuclear magnetic resonance spectroscopy. Biosynthesized 5-IP_7_ was obtained by adapting the protocol reported by Puschmann *et al*. [22], using our in-house prepared LSL-*Hs*IP6K2 and a total reaction volume of 300 mL (see Supplementary methods).

### Protein expression and purification

Codon optimized *Hs*DIPP1 (residues Met1-Ser148) and the mutants presented in this work were cloned in a pET28a(+)-His-TEV vector (Genscript) and expressed in *E. coli* BL21 Star. The cells were transformed and grown in 2TY medium supplemented with 50 µg/mL kanamycin at 37 °C until an OD600 = 0.7-0.9. Protein overexpression was induced with 0.4 mM IPTG and incubation for 16 hours at 16 °C. For cell lysis, the cells were resuspended in buffer A (20 mM Tris/HCl pH 7.5 [4 °C] and 500 mM NaCl) and sonicated. Clarified cell lysate was loaded onto a 5 mL HisTrap™ HP column (Cytiva) previously equilibrated with buffer B (20 mM Tris/HCl pH 7.5 [4 °C], 300 mM NaCl, 40 mM imidazole). Protein elution was performed with a 100 mL gradient of 0.04 – 1 M imidazole. The sample was diluted 1:6 in buffer C (20 mM Tris/HCl pH 7.5 [4 °C], 1 mM DTT) and subsequently loaded onto a 5 mL HiTrap™ Heparin HP column (Cytiva) pre-equilibrated with buffer D (20 mM Tris/HCl pH 7.5 [4 °C], 50 mM NaCl, 1 mM DTT). Elution was performed with a salt gradient of 0.05–1 M NaCl in 100 mL and His-tag was removed by incubating overnight at 4 °C with in-house produced TEV (tobacco etch virus) protease at a mass ratio of 1:40 (TEV/protein) in some of the samples used. Subsequently, only the samples incubated with TEV were loaded onto a 1 mL HisTrap™ HP column (Cytiva) equilibrated with buffer B and eluted at 40 mM imidazole. Finally, the protein sample was passed through a 16/600 Superdex75 gel filtration column (GE Healthcare) equilibrated with buffer E (20 mM Tris/HCl pH 7.5 [4 °C], 150 mM NaCl, 1 mM DTT). Each sample was analyzed by SDS-PAGE and concentrated to approximately 20 mg/mL, except for ΔN-DIPP1, which was concentrated to 1 mg/mL. Finally, the samples were stored at -80 °C until use.

Wt-*Sc*DDP1 and Δnose-*Sc*DDP1 samples were expressed and purified as reported previously [16].

### Protein crystallization and structure determination

*Hs*DIPP1 samples were submitted to different commercial screens (JCSG++, JBS and PACT++ from Jena BioScience) using an Oryx robot (Douglas Instruments) and sitting drop vapor diffusion method at 18 °C. Different crystallization conditions were identified for the *Hs*DIPP1 constructs, most of them consisting on 20-32% PEG 6K, 0.1 M sodium acetate pH 5.0, 0.2 M LiCl, and 1-10 mM MgCl__2__ (Table S1). In other cases, NaF was added, or MgCl__2__ was replaced with MnCl__2__. Other complexes were obtained under the different conditions included in Table S1. The crystallographic data sets for the complexes were collected at 100K at the ALBA and ESRF synchrotrons. Data were processed automatically using autoPROC [23] and XIA2_DIALS by the Synchrotron Facility. Some datasets were processed and anisotropically truncated with STARANISO (local ⟨I/σ(I)⟩ ≥ 1.20); for these datasets, directional resolution limits as well as ellipsoidal and spherical completeness are reported. The electron map of the DIPP1-R89S/PCP-IP_8_ complex in 5PP mode suggests that the 1-PP moiety of the PCP-IP_8_ displays a more flexible interaction within the active site likely attributable to the absence of Arg89. Molrep [24] was used for structural solution and Refmac5 [25] for coordinates refinement, which was alternated with model building cycles performed in COOT [26]. Final models were validated using the wwwPDB Validation Service. The atomic coordinates and structure factors for the structures reported in this work have been deposited in the Protein Data Bank under accession codes 9T49, 9T4A, 9T48, 9T4B, 9T4C, 9T4D, 9T4G, 9T4H, 9T4I, 9T4J, 9T4K, 9T4L, 9T4M, 9T4E and 9T4F (see Data Availability and Table S1).

### Enzyme assays

Enzymatic reaction products were resolved by HPLC on a CarboPac PA-200 column according to Whitfield *et al*. [27] and detected with ferric ion. Phosphate release assays were performed as described in the Supplementary methods, and aliquots of the products were analyzed by suppressed ion-conductivity on a Dionex (UK) ICS-2100 system after resolution on a 250x2 mm AS11 (Dionex) column with 50x2 mm AG11 (Dionex) guard column. Full details of the procedures are provided in the Supplementary methods.

### Differential Scanning Fluorimetry (thermal shift experiments)

The thermal denaturation profiles of *Hs*DIPP1 and its mutants were obtained by measuring the intrinsic fluorescence spectra of the proteins as a function of temperature at wavelengths of 330 and 350 nm using the Tycho NT.6 system (NanoTemper Technologies). The Tycho NT.6 software generates the denaturation curves based on the 350/330 nm ratio and automatically calculates an inflection temperature (Ti) value. For the assay, 10 µL samples were loaded into small glass capillaries and heated from 35 °C (308 K) to 95 °C (368 K) at 30 ºC per minute. Integrity of the samples at the ramp initial temperature was confirmed by comparing fluorescence spectra at 20 °C and 35 °C independently measured (FLUOstar Omega, BMG Labtech). Experiments were carried out in triplicate, and data presented are the average ± SD. All measurements were carried out from a mixture of DIPP1 diluted to a final concentration of 10 µM in 20 mM Tris/HCl pH 7.5 and 1 mM DTT, with (buffer E) and without 150 mM NaCl, with the inositide ligand at 100 µM and MgCl_2_ at the specified concentrations.

### Analytical ultracentrifugation (AUC)

We performed sedimentation velocity experiments in an XLA analytical ultracentrifuge equipped with UV-VIS absorbance optics (Beckman-Coulter Inc.) using 3 mm Epon-charcoal double-sector centerpieces. wt-DIPP1 samples at 155 µM, with and without 5 mM IP_6_, were measured in 20 mM Tris/HCl pH 7.5, 150 mM NaCl and 0.15 mM DTT. Experiments at low ionic strength were performed in 20 mM Tris/HCl pH 7.5, 0.15 mM DTT using the following samples: *His*-DIPP1 at 135 µM, with and without 2 mM and 10 mM IP_6_; DIPP1-R41A at 106 µM, with and without 2 mM IP_6_; DIPP1-N112S at 106 µM with 2 mM IP_6_ and 70 µM without IP_6_; and *His*-DIPP1-R41A-N112A at 114 µM with 2 mM IP_6_ and 92 µM without IP_6_. Samples were sedimented at 48,000 rpm at 6 °C. Sedimentation profiles were obtained by monitoring absorbance at 280 nm. Differential sedimentation coefficient distributions were calculated by least square boundary modelling of the data using the c(s) method by SEDFIT [28] and sedimentation values corrected to standard conditions (*s*_20,w_; 20 ºC, water, and infinite dilution) using SEDNTERP software.

### Dynamic light scattering (DLS)

DLS experiments were performed in a DynaPro Titan instrument (Wyatt Inc), at 20 ºC. 100 µM wt-DIPP1 samples were in 20 mM Tris/HCl, pH 7.5, 0.15 mM DTT, with and without 5 mM IP_6_, previously filtered (0.1 μm, Anotop 10 Plus filters, Whatman) and centrifuged for 30 min. Experiments aimed to determine the effect of the ionic strength and of lower IP_6_, closer to physiological concentrations, were conducted with 50 µM wt-DIPP1 samples in 20 mM Tris/HCl, pH 7.5, 0.15 mM DTT, with and without NaCl, or with and without 100 µM IP_6_, filtered and centrifuged as before. Autocorrelation profiles were exported using Dynamics V6 software and analyzed using user-written scripts and functions in MATLAB (version R2025b, Mathworks, Natick, MA) as described elsewhere [29] to get the apparent diffusion coefficient (*D*_app_) values and the uncertainties, estimated by least-squares modeling using a modified sum-of-squares profile method [30]. Each independent replicate is the average of 8-44 acquisitions. Reported values from analysis to a single species are apparent diffusion coefficients ± SD representing confidence limits at 68%. Data analysis of the samples in the presence of IP_6_ evidenced the presence of a faster diffusion species, corresponding to a discrete major species, and a second species with substantially slower diffusion, corresponding to higher order species and contributing to a lower extent to the whole sample population. A compatible double exponential decay model was fit to the data with 5mM IP_6_, using as starting values for the translational diffusion coefficient of the higher order species that retrieved by the analysis in the absence of IP_6_. Diffusion coefficient reported for the discrete species is the average of the diffusions from individual analysis of 4 replicates (average of 5-11 acquisitions each) ± SD calculated by error propagation.

The apparent molar mass of DIPP1 discrete species was calculated using the Svedberg equation [29] from its *s*_20,w_ and *D*-values independently measured by sedimentation velocity and DLS, respectively.

### Molecular docking

Docking experiments were performed with GOLD [31] and visualized with Hermes programs (CCDC Software Ltd.) using different input protein (PDB codes 6pck, 6pcl, and the coordinates of DIPP1/PCP-IP_8_ complexes obtained in this work) and IP_8_ coordinates (built to mimic the conformations of PCP-IP_8_ obtained in 1PP-SP and 1PP-TB modes, as well as that minimized with Mercury [32] conformer generation option). In all cases we chose the default ChemPLP function and added hydrogen atoms to the ligands. The active site was dimensioned to 4 Å around the ligand in each protein complex, and we searched for 10 solutions in each ligand run, allowing early termination when 3 identical solutions with a r.m.s. below 0.5 Å^2^ were found. Subsequently, we performed multiple runs varying several parameters. Thus, we considered retention of two or three Mg^2+^ ions (depending on the input coordinates) and inclusion or exclusion of water molecules mediating protein–ligand contacts. When waters were included, we allowed free rotation, translations up to 1.5 Å, and toggled their presence (on/off). Initially, the protein was kept rigid and the ligand at default flexibility. Next, we allowed flexibility in some protein active site residues, whereas unfavorable ligand conformations made it necessary to constrain torsions in the inositol ring substituents (either the first six exocyclic C–O bonds, the first twelve exocyclic C–O–P bonds, or all torsions). Additionally, in some runs we enabled the ring-flip option, which allowed interconversion between chair and boat conformations. Although multiple settings reproduced poses obtained experimentally, our best-performing ones retained two Mg^2+^ ions, constrained twelve (or all) torsions in the inositol substituents without ring flipping, and allowed side-chain flexibility for Arg41 and Arg89 residues, in accordance with their strategic position in the active site and experimentally observed flexibility.

### Conformational sampling

The energies of IP_8_ conformations were calculated using RDKit, an open-source cheminformatics software [33]. The initial structure of IP_8_ was built from the PCP-IP_8_ model obtained in this work and transformed to a mol2 file with Mercury [32]. Multiple conformations of IP_8_ were generated using RDKit's EmbedMultipleConfs function and ETKDG (Experimental-Torsion Knowledge Distance Geometry) method. The following parameters were used: numConfs=50 to generate 50 conformations, pruneRmsThresh=0.5 to ensure conformational diversity, and maxAttempts=1000 to increase the likelihood of successful embedding/valid conformation. Each generated conformation was subjected to energy minimization using the Universal Force Field (UFF) implemented in RDKit, with the UFFOptimizeMolecule function. The optimization criteria included a maximum of 1000 iterations/cycles and an energy convergence threshold of 0.001 kcal/mol. The energy of each optimized conformation was calculated using the UFF. Energies were compared to identify unique conformations based on a threshold of 0.1 kcal/mol to filter out duplicates. Software and Tools: RDKit Version: 2024.03.5; Python Version: 3.12.4; CCDC: 2023.1.0.

### Molecular dynamics simulations

In order to perform molecular dynamics with the wild-type enzyme and its natural substrate IP_8_, we produced DIPP1-IP_8_ structures by modelling the IP_8_ ligand onto the electron density map of its non-hydrolyzable analogue in DIPP1-R89S/PCP-IP_8_ dataset by reverting the R89S mutation. Molecular dynamics were performed with AMBER22 [34] in the Galician Supercomputing Centre (CESGA). Prior to the simulations, the system was prepared as follows: i) IP_8_ was parametrized with the GAFF2 force field and DIPP1 with the ff19SB force field, and the three active site Mg^2+^ ions were maintained in the input structure, whereas water and other ions were removed, ii) missing hydrogens were added with Open Babel [35] and protonation states were assigned for pH 7.0, iii) the complex was solvated in a truncated octahedral box of TIP3P water with a 12 Å buffer and the system was neutralized by adding Na+/Cl- counterions. After the preparation step, the system was minimized first with positional restraints and then unrestrained. Then the system was heated (0 to 300K over 50 ps) using a Langevin thermostat and then equilibrated at 300 K and 1 atm for 100 ps using a Berendsen barostat. Distance-based NMR-type restraints were used along these preparation steps to maintain the structure as closed as the experimental one. Finally, production simulations (3 x 100 µs) were performed in the NPT ensemble (300 K, 1 atm) using the Berendsen barostat (taup = 5.0 ps) and Langevin thermostat (γ = 1.0 ps^−1^). Active site Mg^2+^ ions were restrained using NMR-type restraints to their coordinating residues in the protein to stabilize their configuration during the simulation. Cremer-Pople rings puckering were calculated with the CPPTRAJ [36] utility of AMBER22 [34] and hydrogen bonds were analyzed with VMD [37].

## Results

### Twist-boat and chair conformations for IP_8_ 1-PP and 5-PP hydrolysis respectively

**Fig. 1:**
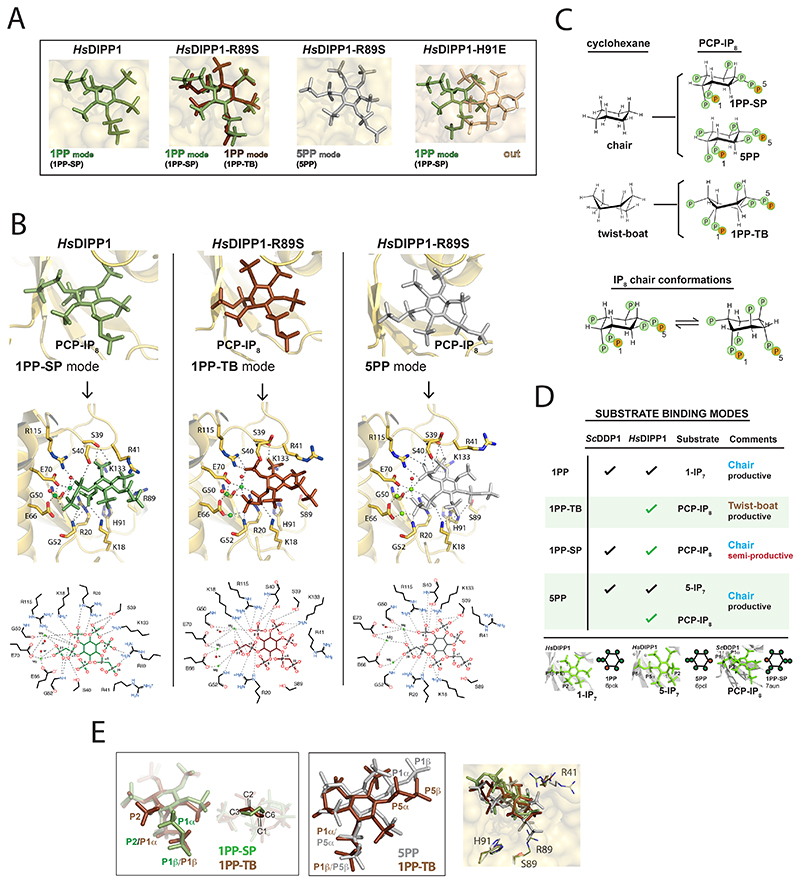
*Hs*DIPP1 substrate binding. (A) *Hs*DIPP1 structures in the presence of PCP-IP_8_ obtained in this work, showing its binding modes: *Hs*DIPP1 in the 1PP-SP mode; R89S mutant in 1PP-SP and 1PP-TB modes and R89S mutant in 5PP mode; and H91E mutant in the 1PP-SP mode and also a secondary position outside the active site similar to that reported previously [14]. The selected color code for PCP-IP_8_ is green, brown and grey for 1PP-SP, 1PP-TB and 5-PP modes, respectively. (B) (Top) PCP-IP_8_ binding to *Hs*DIPP1 (yellow cartoons) in the 1PP-SP, 1PP-TB and 5PP modes, represented by green, brown and grey sticks respectively. (Middle) Structure of the enzyme active site (yellow cartoons and sticks) showing residues interacting with the substrate in each mode; green and red balls depict magnesium and water, respectively. (Bottom) 2D representation of interactions shown in the middle row. (C) Two-dimensional representations of chair and twist-boat conformations of cyclohexane (left), compared with ChemDraw images of PCP-IP_8_ conformations obtained from the coordinates of *Hs*DIPP1 crystal structures, exported as .mol files. PCP-IP_8_ adopts a chair conformation in the 1PP-SP and 5PP modes, whereas in the 1PP-TB mode it resembles a twist-boat conformation. At the bottom, an equilibrium between both chair conformations of IP_8_ is shown, obtained from conformers generated by Mercury. Phosphate groups are represented as green circles, whereas the hydrolysable β phosphates are shown in orange. (D) Schematic table showing the different binding modes identified for *Hs*DIPP1/*Sc*DDP1 substrates 1-IP_7_, 5-IP_7_, and the analogue PCP-IP_8_, including those modes reported previously (black check marks) and those identified in this work (green check marks), with a summary of their main features. The images below illustrate the productive binding modes previously determined for 1-IP_7_ (1PP-mode) and 5-IP_7_ (5PP mode) in *Hs*DIPP1 (left and middle, respectively), and the semi-productive mode determined for PCP-IP_8_ (1PP-SP mode) in *Sc*DDP1 (right). Schematics of the inositides are shown, where the green circles represent phosphate groups, and the axial substituent at position 2 is highlighted in red to illustrate the different orientations of the inositide ring plane among the previously known binding modes. (E) Superposition of binding modes: left, 1PP-SP (green) and 1PP-TB (brown), as found in the *Hs*DIPP1 active site, illustrating the conformational change caused by C6-C1-C2-C3 ring torsion, and middle, for 5PP (grey) and 1PP-TB (brown) modes. The rightmost panel shows superposition of the three productive modes, and the residues Arg41, His91 and Arg/Ser89 in each of them.

In order to capture unreported *Hs*DIPP1 binding modes for its substrate IP_8_ (Fig. S1), we prepared crystals of wild-type *Hs*DIPP1 (wt-DIPP1) and several mutated *Hs*DIPP1 versions (Fig. S2); DIPP1-R41A, -R89S, -H91E, -H91M, -E108N and -N112S; in the presence of the synthetic and non-hydrolyzable analogue PCP-IP_8_ (Table S1). These residues were chosen for mutation due to their strategic position and all of them retain the ability to bind the IP_8_ analogue (see below). Although several hundred crystals were analyzed, the ligand electron density maps obtained were consistent with a mixture of PCP-IP_8_ binding modes in most of the cases. We selected a few crystal complexes with the less complex scenario (Figs. 1A and S3), obtained with wt-DIPP1 or mutants R89S and H91E. They allowed PCP-IP_8_ modeling in four different modes, three of them in the catalytic site (Fig. 1B). Among the latter, we observed two binding modes where the 1-PP moiety is positioned near the catalytic residues (1PP modes), and another mode where the 5-PP moiety occupies that position (5PP mode). These modes are associated with two distinct conformations of the inositol ring, the canonical chair, with five equatorial and one axial substituents, and a twist-boat conformation (Fig. 1C). One of the 1PP modes, the one most frequently captured in our crystals, using either wt- or mutated *Hs*DIPP1, adopts a canonical chair conformation (Fig. 1B-left and 1C). This mode is equivalent to the one previously reported in *Sc*DDP1 [16], which we refer to as the “semi-productive mode (1PP-SP mode)”. In this mode, the diphosphate moiety does not show the typical fit of a PP moiety in Nudix enzymes; though Pβ is well positioned, Pα is displaced. The other 1PP mode, captured mainly in DIPP1-R89S crystals in mixed forms (Fig. 1A), features an unexpected twist-boat conformation, herein named the 1PP-TB mode (Figs. 1B-middle and 1C). This mode places the 1-PP group in a catalytically productive orientation. Notably, a similar mixture of PCP-IP_8_ modes (1PP-SP and 1PP-TB) is observed in several wt-DIPP1 crystals (Fig. S3E). However, the 1PP-SP mode is predominant, precluding a stable refinement of the wt-DIPP1 structure including the 1PP-TB mode. To our knowledge, this is the first reported structure in inositol polyphosphates (InsP) metabolism providing evidence of an InsP adopting a twist-boat conformation. Finally, we modeled the 5PP mode in the other DIPP1-R89S crystal. This represents the first structure showing an IP_8_ analogue bound in this mode, which is in turn catalytically productive and adopts a canonical chair conformation (Figs. 1B-right and 1C). The 1-diphosphate moiety remains noticeably disordered in the crystal (Fig. S3C), likely due to the R89S mutation. Remarkably, both productive binding modes show three Mg^2+^ ions, whereas the SP mode presents only two (Fig. 1B).

That *Hs*DIPP1 and its orthologue *Sc*DDP1 bind substrates in different modes has been reported previously [14,16,38] (Fig. 1D). Prior to this work, it was established that 1-IP_7_ and 5-IP_7_ substrates bind in productive modes for 1-PP and 5-PP hydrolysis respectively [38] (Figs.1D, bottom-left and middle), both in canonical chair conformation. Thus, the productive 5PP mode is equivalent to the one described here for IP_8_ binding. By contrast, the 1PP mode is not, since 1-IP_7_ can achieve a productive binding without distorting the canonical chair conformation. As mentioned above, PCP-IP_8_ had previously been captured only in the semi-productive mode in *Sc*DDP1 (Fig. 1D, bottom-right). The observation of this binding mode in *Hs*DIPP1, in the present work, (Figs. 1A and 1D), reinforces its relevance. The identification of two productive modes for IP_8_ binding completes the current model of substrate binding modes in the DIPP1 family (Fig. 1D).

Similarities and differences in IP_8_ binding modes and their recognition by the enzyme are illustrated in Fig. 1E. Both 1PP modes, 1PP-SP and 1PP-TB, are equivalent, changing mainly the position of atoms C1 and C2 of the ring with the subsequent torsion modification (Fig. 1E, left). Therefore, the 1-PP moieties and their contiguous phosphates show changes in their recognition. The opposite is observed when comparing both productive modes, 1PP-TB and 5PP (Fig. 1E, middle), which share binding to the hydrolyzing PP moiety and its surroundings. Three active site residues, Arg41, Arg89 and His91, surround the most variable inositide-binding region across the productive IP_8_ binding modes proposed (Fig. 1E, right). Arg41 is highly flexible; it aligns with the inositide ring and it is next to a phosphate group in all binding modes except the 5PP modes. In the latter, the axial 2-phosphate requires space, forcing Arg41 and its loop (L1, see below) to shift slightly away from the active site (Fig. S4A). By contrast, Arg89 presents lower flexibility and appears essential across all substrate binding modes as observed in wt-DIPP1 and inferred from the R89S crystals (Fig. 1B). Still, the structural data do not explain why mutation of this residue allowed capture of the TB conformation with increased occupancy. Finally, His91 only interacts with a phosphate in some binding modes and it seems to be aligned to Arg89. Mutagenesis at these three residues, Arg41, Arg89 and His91, significantly impacts enzyme activity and stability, as shown below.

### *In silico* analysis reinforces experimental crystallographic results

**Fig. 2:**
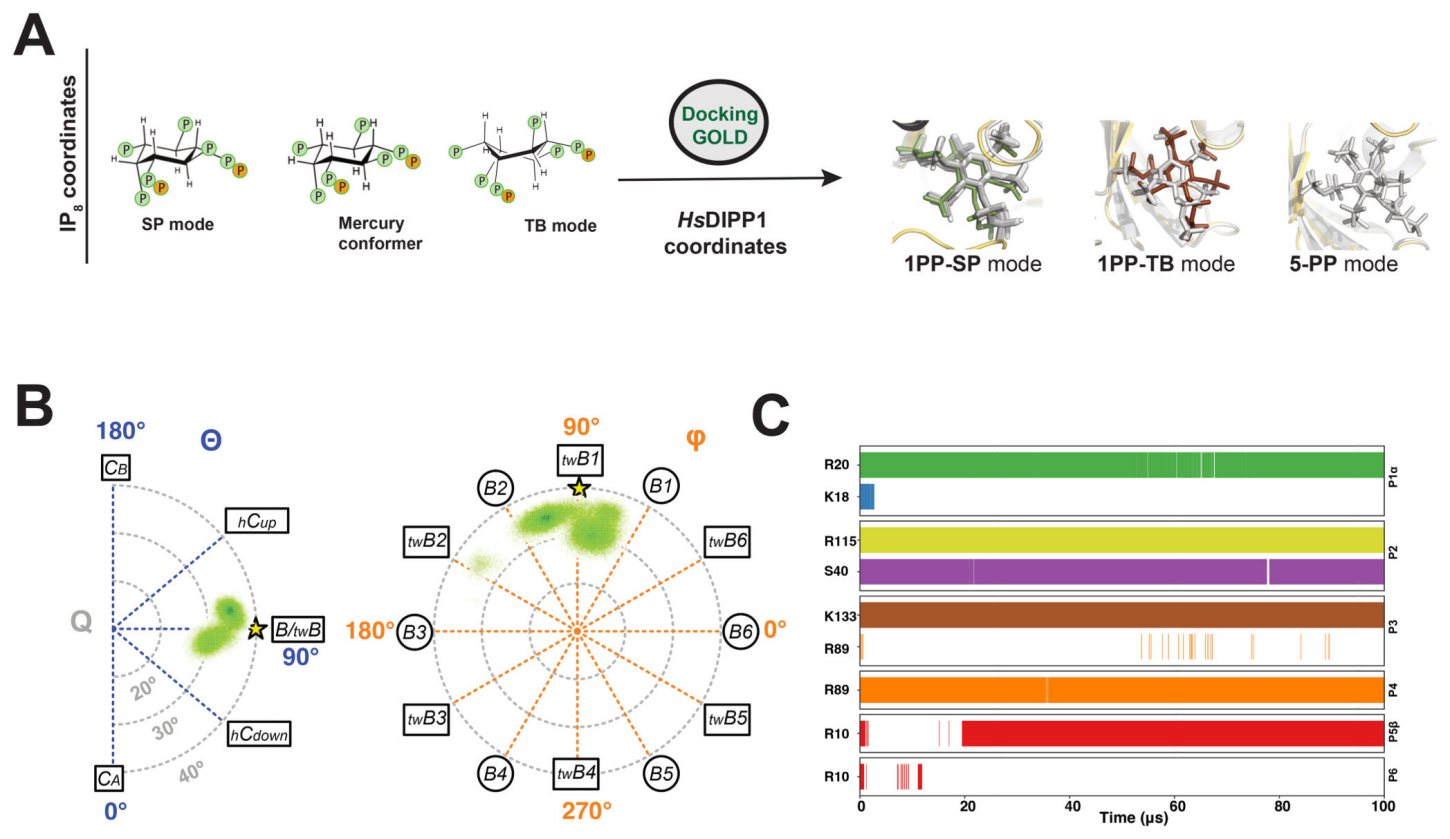
Computational support for IP_8_ binding to DIPP1 (A) Schematic of GOLD docking solutions. Left, 2D representations of IP_8_ used for docking; two chair conformations (from Mercury and *Hs*DIPP1 structures) and one twist-boat conformation (from *Hs*DIPP1 structures). We used *Hs*DIPP1 coordinates from 6pcl, 6pck and those obtained in this study in complex with PCP-IP_8_. Phosphate groups are identified as in Figure 1C. Right, representative solutions obtained (white sticks) and superposed on the experimental complexes (color-coded as in Figure 1A). We reproduced *in silico* the two productive (5PP and 1PP-TB) and the semi-productive (1PP-SP) modes for IP_8_ binding. (B) Polar density distribution maps of the Cremer-Pople pseudo rotational angles Q (grey lines), φ (orange lines) and θ (navy lines) of the IP_8_ ring across the molecular dynamics simulation starting from the 1PP-TB mode conformation (green shades). Yellow start marks the corresponding values for the IP_8_ conformation in the starting DIPP1-IP_8_ complex. Location of the canonical conformations for φ and θ values are labelled (C= Chair, hC= half-hair, B= Boat and twB= twisted-boat). (C) Hydrogen bond events between IP_8_ and DIPP1 selected donor sidechains during one of the productions (data for the other two productions are shown in Fig. S5). Stick graphs are color coded by protein residue.

We performed docking with GOLD [31] using different *Hs*DIPP1 experimental coordinates (Figs. 1B and 1D, see Materials and methods) and three different IP_8_ coordinate sets. These include two InsP_8_ in the chair conformation, equivalent to those observed in 1PP-SP mode complexes and to the one generated computationally, and one in the twist-boat conformation as found in the 1PP-TB mode complex (Fig. 2A, left). The program returned poses that predicted IP_8_ binding to *Hs*DIPP1 in the three modes observed here experimentally: the two productive binding modes (1PP-TB and 5PP), and the semi-productive 1PP-SP mode (Fig. 2A, right), also reported for PCP-IP_8_ in Marquez-Moñino *et al* [16]. Overall, the number of chemically sensible poses increased as the ligand bond-fixing criteria were tightened. Accordingly, the highest prediction rates were observed when 12 or all the inositide torsions were fixed (see Materials and methods). Under those settings, all three modes (5-PP, 1PP-SP and 1PP-TB) were predicted, each reaching up to 100% of poses in selected independent runs. Notably, the 1PP-TB mode is predicted both when the supplied IP_8_ coordinates were in TB conformation (all torsions fixed) or from the chair conformation (ring flipping allowed), but in the latter case only in 10% of poses.

Furthermore, we performed preliminary energy calculations for different IP_8_ conformers. Thus, we obtained 50 energetically and geometrically feasible conformations using RDKit [33], including two chair and several twist-boat conformations (Table S2). As expected, the more stable forms (chair 1 in Table S2) are similar to that observed in 1PP-SP and 5PP modes, excluding the conformations of the diphosphate β-phosphate groups. Notably, five conformations out of 50 closely resemble the twist-boat conformation observed in the X-ray crystallographic structure showing the 1PP-TB mode. The IP_8_ twist-boat conformation is predicted to be 4-8 kJ/mol less stable than the chair ones, but it is still considered viable according to RDKit calculations. These results should be interpreted qualitatively, since protonation states of the phosphate groups and ionic effects such as stabilization by Mg^2+^ were not considered.

Finally, we performed molecular dynamics simulations with AMBER on *Hs*DIPP1-IP_8_ complexes to provide additional support for the 1PP-TB binding mode of the wt enzyme with the native substrate. To this end, we modeled in the wt-DIPP1 structure the IP_8_ in the twist-boat conformation with the protein containing 3 Mg^2+^ ions, as found in the 1PP-TB mode complex. The positions of the Mg^2+^ ions were restrained. Productions were extended for 100 µs and trajectories were analyzed with the Cremer-Pople ring puckering parameters for the IP_8_ ring [39] and the evolution of IP_8_-protein hydrogen bonds (Fig. 2B, 2C, S5 and Supplementary Movies 1-3). The productions (300 µs in total) sample several conformations around the twisted boat 1 conformation observed in the DIPP1-R89S complex with PCP-IP_8_. Chair conformation, envelope-like or non-related boats were not explored (Fig. 2B). Furthermore, the ligand recognition sphere is mostly maintained compared to our experimental data (*cf*. Fig. 1B and Fig. 2C and Fig. S5). The simulation suggests that Arg89 could bind phosphates 3 and 4. It also provides hints about the role of Arg10 in P5β and P6 recognition, which were not apparent in the experimental structures due to the participation of Arg10 in crystal contacts.

Altogether, these computational results support our experimental evidence for the DIPP1 substrate binding spectrum (Fig. 1B). Thus, docking calculations reproduce the three binding modes observed experimentally (Fig. 2A), whereas energy calculations identify both IP_8_ chair conformations, followed by twist boat conformations, among the 50 most stable conformers (Table S2). Finally, molecular dynamics simulations further support the requirements of IP_8_ to adopt a twist-boat conformation for the hydrolysis of the 1-PP moiety by DIPP1 (Fig. 2B).

### DIPP1 flexibility outlined by different protein-ligand complexes

**Fig. 3:**
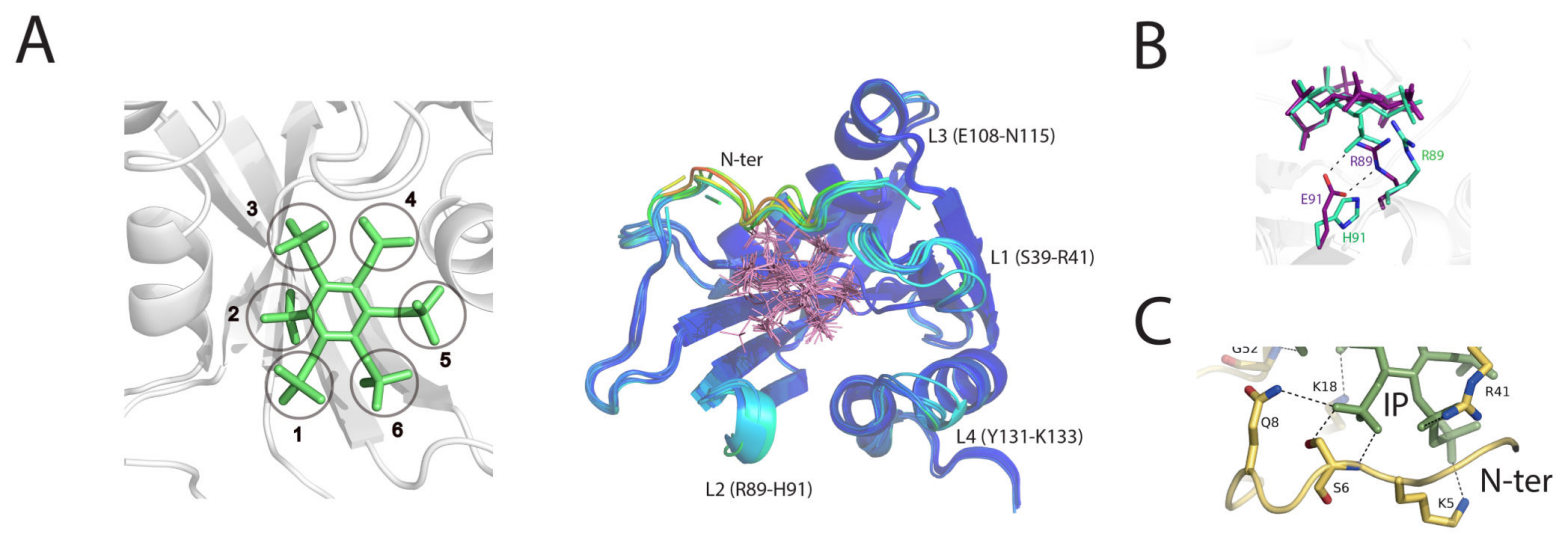
DIPP1 flexibility. Comparison of *Hs*DIPP1-ligand structures. (A) Structure of DIPP1/IP_6_ complex highlighting the six phosphate recognition sites. On the right, superposition of all DIPP1/IP_6_ and DIPP1/PCP-IP_8_ structures obtained in this work, including wt- and mutants R41A, R89S, H91M, H91E, E108N and N112S, showing five regions (N-terminal and loops L1, L2, L3 and L4) with the highest structural variation. The color code for the protein is chosen based upon the thermal B-factors. (B) Superposition of the IP_6_ in wt-DIPP1 (green) and DIPP1-H91E (purple) crystals showing the differences observed in the Arg89 conformation, due to its interaction with Glu91, and in the IP_6_ position. (C) A close-up of the N-terminal (yellow cartoons and sticks) interactions with the substrate (green sticks).

In addition to the *Hs*DIPP1 complexes with PCP-IP_8_ or IP_8_ described above, we crystallized most *Hs*DIPP1 mutants in the presence of IP_6_ to go deeper into the role of the mutated residues in protein function (Table S1). Several modes for product IP_6_ binding have been reported by us and others [14,16], the one predominantly obtained here (Fig. 3A) corresponds to one of these modes. Most mutants show greater disorder at the IP_6_ site than the wild-type, as expected from the removal of residues critical for substrate binding. In spite of this, all mutants bind IP_6_ in the same site only showing small shifts, the most notable observed in the H91E mutant, where IP_6_ rotates and moves about 3 Å away from Glu91 (Fig. 3B). This is due to Glu91 promoting a different rotamer of Arg89 (Fig. 3B), resulting in a pronounced stabilization (~7 ºC) of the mutant in comparison with the wild-type (see below).

An analysis of all the coordinates’ thermal B-factors and their comparison across the different complexes, including those with PCP-IP_8_, reveals that *Hs*DIPP1 shows flexibility in five regions. Three of them are loops involved in inositide binding: Ser39–Ser40–Arg41 (L1, also named here as the serines loop), Arg89–His91 (L2) and the loop that harbors Lys133 (L4) (Fig. 3A, right). Remarkably, some crystal complexes exhibit a large variation in the loop L1 that extends to the following residues, which would form part of the polyphosphate binding site as observed in *Sc*DDP1 [16]. In addition, this loop moves away from the active site in some complexes, being closer in the 1PP modes and farther in the 5PP modes observed in protein-PCP-IP_8_ complexes, as anticipated before (Fig. S4). The two other regions with large mobility are a fourth loop, Glu108-Arg115 (L3) and the N-terminal region (Fig. 3A, right). L3 seems to be involved in both substrate binding and protein–protein association (see below). The crystal structures suggest a role for the N-terminus in substrate binding as this flexible region becomes partially ordered in some crystals, covering the active site (Fig. 3C). To test its possible role, we generated two ΔN-DIPP1 versions starting at residues 8 and 14. Only the former variant, which retains activity against IP_8_ and both IP_7_ isomers (see below), was successfully expressed in bacteria.

### Characterization of *Hs*DIPP1 and its mutants toward PP-InsP substrates

Mutated *Hs*DIPP1 variants were analyzed through differential scanning fluorimetry (Fig. S2D) and activity assays to assess their ability to bind and convert the inositide substrates, relative to the wt-enzyme. We examined mutations at Arg41, Arg89, His91 residues and the N-terminally truncated form (ΔN-DIPP1), implicated in ligand binding as described above. We also targeted residues Glu108 and Asn112 from loop L3 because of their putative role in protein oligomerization or ligand binding, as described in the next section.

**Fig. 4:**
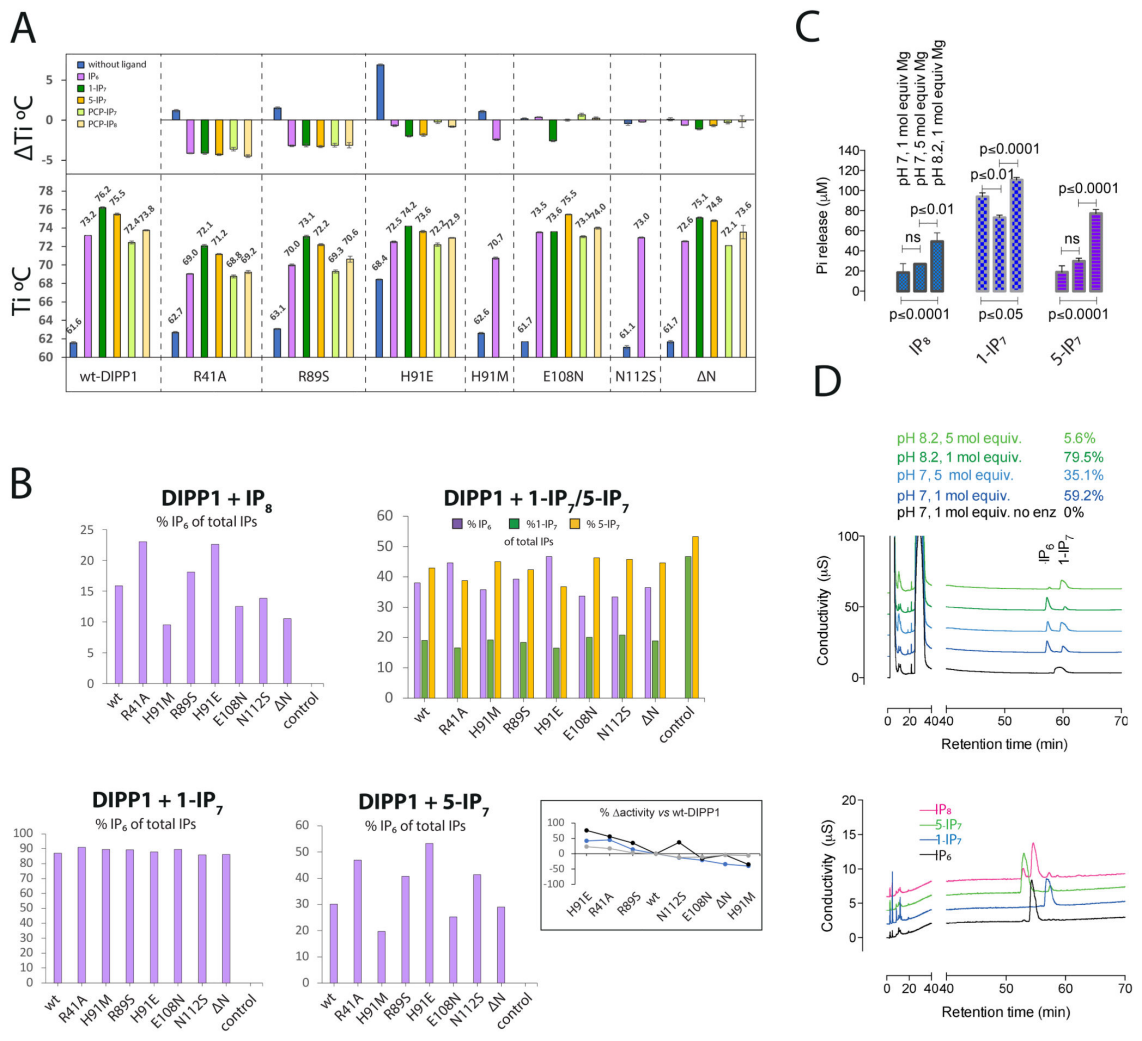
*Hs*DIPP1 samples stability, substrate binding and activity. (A) Analysis of the stability of *Hs*DIPP1 and its mutants produced in this work by thermal shift experiments, showing the Ti values upon binding of IP_6_ and various PP-InsPs and analogues. The corresponding Ti shifts (ΔTi) relative to the wild-type protein are shown above. Data represent means ± SD from three measurements (n=3) using independently prepared samples from the same protein batch. (B) Inositide conversion by wt-DIPP1 and selected mutants analyzed by HPLC (single measurements). Depicted are the amounts of IP_6_ (% of chromatogram total peaks) from hydrolysis after incubation of wt-DIPP1 and the indicated mutants with IP_8_ (top-left), a 1-IP_7_/5-IP_7_ mixture (top-right; amounts of residual IP_7_ isomers also depicted), 1-IP_7_ (bottom-left) and 5-IP_7_ (bottom-middle). Control refers to the measurement in the absence of protein with each specified substrate (starting material in top-right). The squared picture at the bottom-right represents the percentage of activity variation of the mutants *vs*. wt-DIPP1 against different substrates (IP_8_ (blue), 5-IP_7_ (black) and IP_7_ mix (grey)). (C) Wt-DIPP1 phosphatase activities against IP_7_ and IP_8_ substrates. Assays against each substrate were performed under the pH and Mg^2+^ conditions shown above the IP_8_ substrate dataset. Conditions with 5 mol equivalents Mg^2+^ showed precipitation at pH 8.2 but not obviously at pH 7; for all three substrates, Pi release was greatest at pH 8.2 and 1 mol equivalent of Mg^2+^. Means and standard errors of three measurements are shown. For each substrate, the significance or otherwise of differences between means from reactions at different pH/Mg^2+^, as determined by two-way ANOVA, are indicated on the figure. (D) (Top) Suppressed ion-conductivity chromatography of the products of assay with 1-IP_7_ under different pH and Mg^2+^ conditions. Conversion of substrate to product, as %, is estimated from integration of peak areas. (Bottom) Suppressed ion-conductivity chromatography of IP_6_, 1-IP_7_, 5-IP_7_ and IP_8_. Samples made up in water. IP_6_ and IP_8_ coelute.

Wt-DIPP1 thermal stability increased dramatically in presence of its substrate or product and analogues (Fig. 4A and FigS2D). With substrates (1-IP_7_ and 5-IP_7_), but not with the product (IP_6_) or a non-hydrolyzable substrate analogue (PCP-IP_7_), this stabilization decreases in the presence of Mg^2+^ (Fig. S6). We can attribute this to substrate hydrolysis, since thermal stability of the substrate complexes approached that of DIPP1/IP_6_ (Fig. S6A). This is further supported by the trend in the stability shift with increasing concentrations of the cation and with fluoride (Fig. S6B,C), the latter known to inhibit DIPP1 activity [12]. The thermal stability of mutants R41A, R89S, H91E and H91M increased, with different magnitudes compared to the wild-type enzyme (Fig. 4A), likely due to a local decrease in positive charge density. These mutants retained the ability to bind inositides, as proved by their stabilization in the presence of the ligands, but the complexes formed were less stable than the equivalent complexes with wt-DIPP1, as expected for residues directly involved in substrate binding (Fig. 4A). Notably, ΔN-DIPP1 exhibited essentially the same behavior that wt-DIPP1 in thermal shift assays.

Indeed, R41A, R89S, H91E and ΔN-DIPP1 mutants show enzymatic activity towards IP_8_ and both IP_7_ isomers, as do the other listed mutants (Fig. 4B). First, we observe that the three single-point mutants increase the conversion of IP_8_ to IP_6_ relative to wt-DIPP1, with a particularly marked effect in R41A and H91E (Fig. 4B, top-left). By contrast, ΔN-DIPP1 and H91M decrease IP_8_ hydrolysis significantly. However, in none of >30 independent assays with IP_8_ as substrate did we detect an IP_7_ intermediate, regardless of whether the reaction was stopped at low (4%) or high (62%) conversion to IP_6_, suggesting faster hydrolysis of IP_7_ than of IP_8_. Next, to compare hydrolysis of IP_7_ substrates, two experiments were performed. In one, 1-IP_7_ and 5-IP_7_ were mixed in equimolar concentration and presented as substrate (Fig. 4B, top-right). Across the 7 mutants, residual 1-IP_7_ (as % of total peaks in the chromatogram, 46.7% of the starting material) varied between 16.5 and 20.8% (against 19.0% for wt), while residual 5-IP_7_ (46.7% of the starting material) varied between 36.8 and 46.3% (against 42.9% for wt). Here, R41A and H91E were again the most active, giving IP_6_ in 44.6% and 46.7% yield, respectively. In another experiment, single IP_7_ substrates were incubated with enzyme (Fig. 4B, bottom). Across the 7 mutants and wt, conversion to product ranged from 19.7-53.4% for 5-IP_7_ (R41A, 47%; H91E, 53.4%) beside values of 85.8-90.8% for 1-IP_7_. In the latter case, the reactions are most likely substrate-limited. In summary, changes in IP_8_ and 5-IP_7_ hydrolysis show similar trends across DIPP1 variants (wt-DIPP1 and mutants). By contrast, the faster 1-IP_7_ hydrolysis is not substantially affected by these mutations under the conditions tested (Fig. 4B, bottom-left).

As mentioned, Arg41 shifts away upon 5-IP_7_ or IP_8_ binding in the 5-PP mode, accommodating the axial 2-phosphate (Fig. 1B, bottom-right). This would explain why its substitution to Ala increases the rate of 5-IP_7_ hydrolysis. Note that Zong *et al*. [6] reported an increased activity of R41A mutant on both IP_7_s and IP_8_ substrates, though catalytic efficiency was reduced in this mutant. A smaller increase in both IP_8_ and 5-IP_7_ hydrolysis is also observed upon removal of Arg89 side-chain (R89S; Fig. 1B, bottom-right). This may reflect active site enlargement, allowing better accommodation of the axial 2-phosphate. Interestingly, the H91E mutant shows the highest increase in 5-IP_7_ and IP_8_ hydrolysis in spite of introducing a negative charge into the active site. This may result from stabilization of the Arg89 side chain by Glu91 (Fig. 3B). However, ligand-induced stabilization is reduced relative to the wild-type (Fig. 4A), suggesting that H91E mutation could facilitate product release rather than directly enhancing the catalytic step itself. Overall, perturbing these three residues clearly affects IP_8_ and 5-IP_7_ processing.

In the absence of intermediate accumulation (from IP_8_ substrate), comparison of the hydrolysis of IP_8_, 1-IP_7_ and 5-IP_7_ by wt-DIPP1 was made by a phosphate release assay (Fig. 4C). Again, 1-IP_7_ was the best substrate, consistent with previous studies [2,14]. At pH 8.2 and 1 mol equivalent Mg^2+^, phosphate release was increased (compared to pH 7). Interestingly, at increased Mg^2+^ concentration, Pi release from 1-IP_7_ is reduced. For 1-IP_7_, but not for 5-IP_7_ or IP_8_, we were able to resolve the IP_6_ product from the substrate by ion-chromatography with suppressed ion-conductivity detection (Fig. 4D). The ratio of product to substrate was consistent with the amount of Pi liberated under the different assay conditions, with higher Mg^2+^ reducing product formation at pH 7. Significantly, at pH 8.2 with 5 mol equivalent Mg^2+^, we observed (visually) precipitation with all three substrate samples and markedly so for IP_8_. Light scattering and ion chromatography experiments confirmed that at pH 8.2 and 200 µM substrate, 5 mol equivalent of Mg^2+^ precipitates IP_6_ and 1-IP_7_. (Fig. S7). Therefore, the reduced apparent conversion at high Mg^2+^ is largely attributable to substrate/product depletion rather than enzymatic inhibition.

Kurz *et al*. [17] showed that the pH and Mg^2+^ quantity could alter the equilibrium of 1ax/5eq and 5ax/1eq chair conformations for all three substrates and most markedly so for IP_8_, and that more extreme conditions (higher pH and elevated [Mg^2+^]) favor the flipped state. Unfortunately, our Pi-release assay (Fig. 4C) under conditions of variant pH and Mg^2+^ content revealed that for all three substrates, the condition most likely to increase the proportion of substrate in the 5ax/1eq chair conformation (pH 8.2, 5 mol equivalent Mg^2+^) resulted in visible precipitation. However, two observations were made under conditions in which substrate precipitation did not interfere with the analysis: (1) the stability of DIPP1 complexes showed a clear dependence on Mg^2+^ concentration, in association with modulation of hydrolysis (Fig. S6), consistent with the Mg^2+^-dependent changes in PP-InsP binding affinity to wt-DIPP1 reported by Zong *et al*. [14]; and (2) at 1 equivalent of Mg^2+^, activity against all substrates was maximal at pH 8. Although the pH-dependent increase in activity may reflect changes in ionization of the enzyme active site, substrate conformational effects might also be contributing to these observations. The complex interplay of substrate conformation and requirement for metal co-factor in the ternary interaction of protein, substrate, and metal will require further analysis.

### Insights into DDP1/DIPP1 family regulation

#### Crystallographic evidence for DIPP1 self-association

**Fig. 5:**
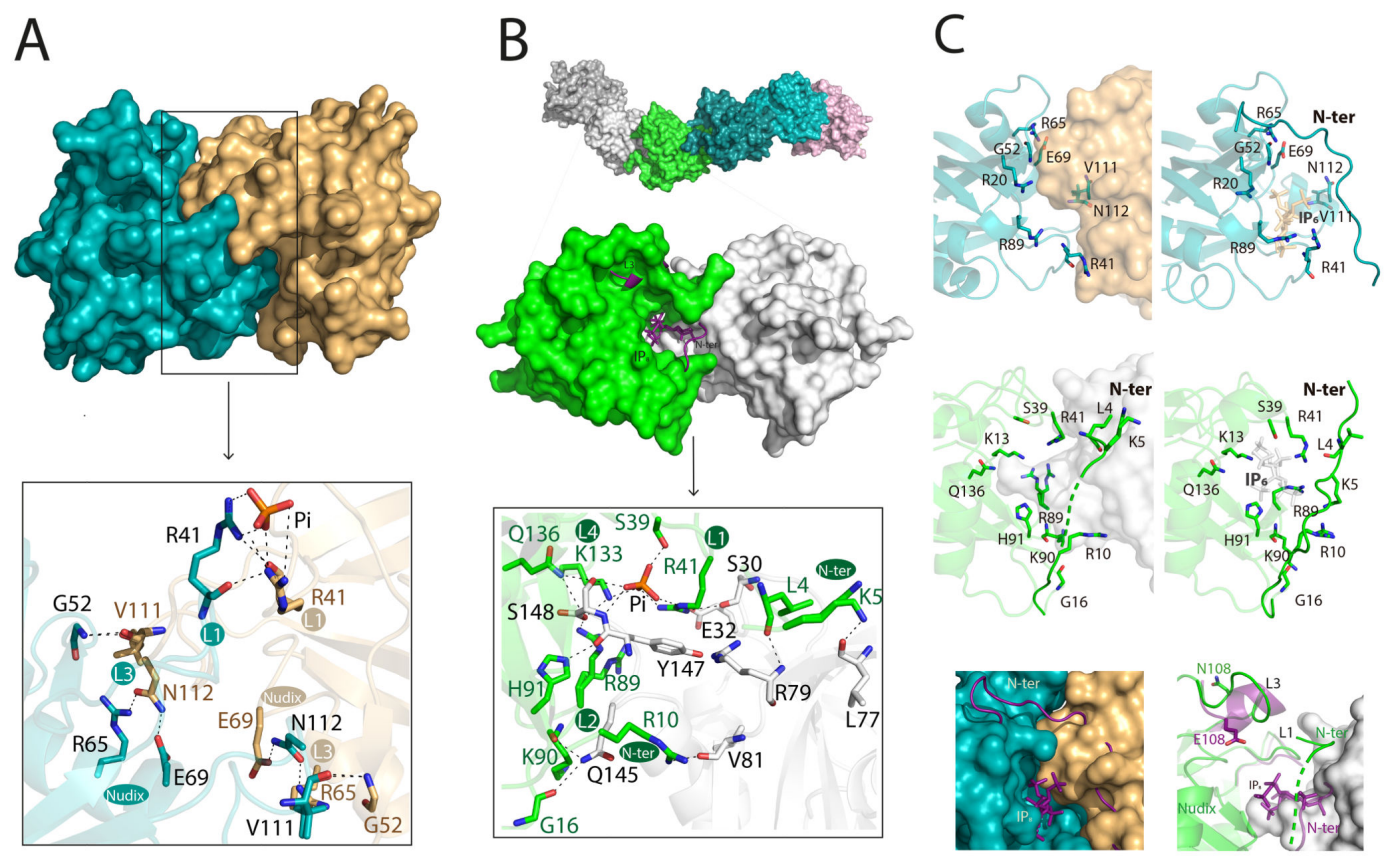
DIPP1 trend to associate in dimers and higher-order oligomers. (A) Surface representation of dimers as found in apo wt-DIPP1 crystals at low salt concentration and, below, a close-up view of the polar interactions observed at the interface, with chain A in teal, chain B in light-orange and the phosphate ion as orange stick. The relevant elements in the structure are highlighted (L1, L3 and Nudix motif). (B) Surface representation of the oligomers formed in apo DIPP1-E108N crystal with a zoom-in on two subunits. The latter is superposed onto the substrate-bound structure (purple cartoons and sticks), displaying the lack of space for the N-terminus and the inositide, as well as the conformational differences in the L3 loop. Below, close-up of polar interactions at the interface, with chain A in green, chain B in white and the phosphate ion as orange stick. (C) Face-to-face comparison of the subunits shown in panels A and B (left, top and middle, respectively) with the IP_6_ bound structure (right, top and middle), conserving the same color scheme. The subunit common to both the self-associated and IP_6_-bound states is shown as a cartoon, whereas the partner subunit (left) or IP_6_ (right) is shown as a surface or sticks representation, respectively. These comparations highlight steric incompatibility between protein association and IP_6_ binding, which also extends to the conformation of the N-terminal region. At the bottom, the two associated states are superposed with the substrate-bound structure, which is shown as purple cartoons and sticks for clarity. In both cases, the inositide-binding region is incompatible with the oligomeric interfaces. Moreover, at the interface between two consecutive monomers in the fiber-like assembly (right), notable conformational changes are observed in L1, L3, and the N-terminal region.

Getting this family to crystallize in the absence of ligands is quite challenging, due to their highly basic active site that leads to a huge protein stabilization in the presence of the highly electronegative substrates or analogues. Previous success in this regard and with the yeast homologue was achieved with mutated versions that diminished the high positive charge in the protein active site [16]. For the present work, we were able to crystallize *Hs*DIPP1 in the absence of ligands (Table S1, Fig. 5), resulting in two different ways of active site occlusion involving *Hs*DIPP1 association in dimers or higher-order states only observed in the apo form crystals.

First, wt-DIPP1 crystals obtained in the absence of ligands revealed a symmetric dimer stabilized by an interface of 729 Å^2^ (buried surface area of 16% of the total surface), and 8 hydrogen bonds (Fig. 5A). At the interface, Arg41 from both subunits interact in a side-main chain fashion and further through a phosphate ion (Fig. 5A). Other residues that seem important for the dimer formation are the key Arg65(A) and Glu69(A) (from chain A) from the Nudix motif in the active site. Both residues form hydrogen bonds with Asn112(B) (from chain B). The latter is part of the mobile region L3 that also contains Arg115, a residue important for substrate binding (Fig. 1B). Mutation of Asn112 does not significantly affect protein stability or stabilization upon IP_6_ binding and it only modestly affects hydrolysis of IP_8_ and 5-IP_7_ (Figs. 4A and 4B). This could be precisely due to the location of L3 next to L1 and the Nudix motif, essential for ligand binding and protein activity. The described protein-protein arrangement is not compatible with substrate binding (Fig. 5C, top and bottom-left).

By contrast, DIPP1-E108N crystals obtained in the absence of ligands revealed a different type of subunits interactions in a head-tail fashion along the crystal (Fig. 5B). In this case, every two subunits interact through an interface of 880 Å^2^ and presenting 11 polar interactions, a number higher than in the previous case (Fig. 5B). Notably, a phosphate ion in the interface also reinforces the interaction. In the interface, Arg41(A) plays an essential role by making hydrogen bonds with Glu32(B) and Ser30(B) as well as cation-π interactions with Tyr147(B). Other important interactions for interface formation are those established between Leu4(A)-Arg79(B) through their main chains; Lys5(A)-Leu77(B) main chain; Arg10(A)-Val81(B) main chain; Arg89(A)-Ser148(B); Lys90(A) main chain and Gly16(A)-Gln145(B); His91(A)-Y147(B) main chain; Lys133(A) and Gln136(A)-Ser148(B). In addition, residues Arg41(A), Ser39(A) and Lys133(A), interact with Ser148(B) through the phosphate ion.

A dramatic change in L3, which contains the mutated Glu108, is observed in the fiber-like DIPP1-E108N (Fig. 5C, bottom-right), relative to all the crystals obtained in presence of ligands (either wt-, E108N or other mutants). As mentioned, this loop, located between the Nudix motif and the L1 (Fig. 5C), is essential to maintain the proper configuration of the DIPP1 active site (and hence ligand binding). The thermal stability of DIPP1-E108N is generally similar to that of wt-DIPP1 in the absence of ligands, but is somewhat decreased in the presence of 1-IP_7_ (Fig. 4A). Similarly, HPLC-monitored enzymatic activity shows that this mutant is active, though the hydrolysis of 5-IP_7_, and more so IP_8_, is slightly decreased, probably due to its strategic position, as was the case for Asn112. Again, this protein-protein arrangement is not compatible with substrate binding (Fig. 5C, middle and bottom-right)

In summary, both ligand-free structural arrangements described above (wt and DIPP1-E108N) are incompatible with substrate binding (Fig. 5C), consistent with the presence of essential substrate-binding residues at the protein-protein interface. Moreover, the N-terminus must be displaced or change conformation (Fig. 5C, bottom) for DIPP1 to self-associate, suggesting a possible additional role of the N-terminus in protein oligomerization. Finally, L3 also appears to be involved in protein association by direct interfaces contact (wt-dimers) or by conformational change (E108N-fibers), with residues from these loops having greater impact on activity than some of the active site residues.

#### Evidence for DIPP1 family association in solution

**Fig. 6:**
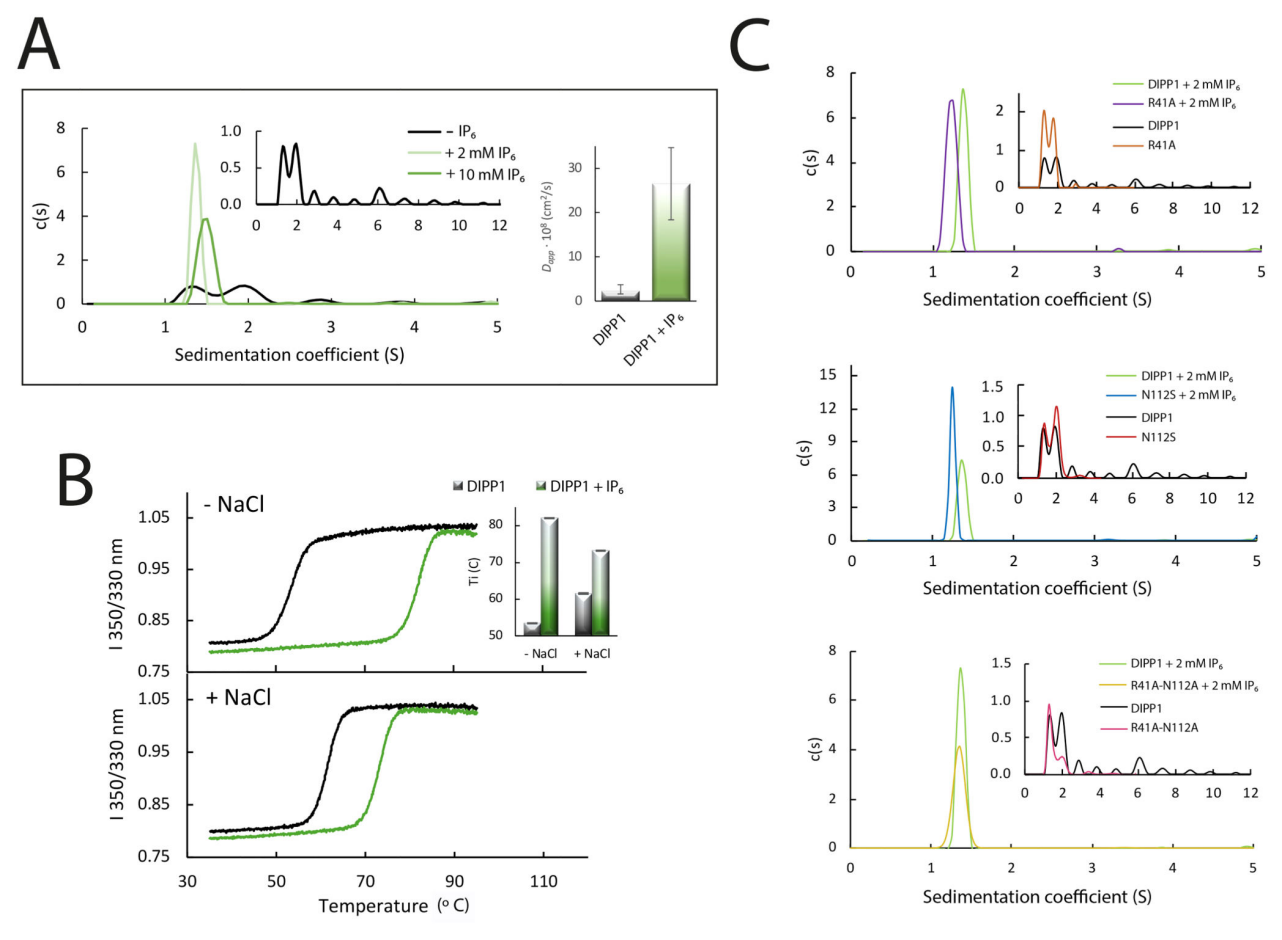
Modulation of DIPP1 association state by IP_6_ in solution. (A) Sedimentation profiles for *His*-DIPP1 in absence and presence of the product IP_6_ at the specified concentrations. Inset shows the whole sedimentation range evidencing the presence of higher order species in the absence of IP_6_. On the right, *D*_app_ values of wt-DIPP1 measured by DLS, representing the overall behavior of the species present. (B) Thermal denaturation profiles of wt-DIPP1 samples (10 μM), represented as the dependence of the 350/330 nm fluorescence intensity ratio over increasing temperature, to show the influence of adding IP_6_ (100 µM) and NaCl (150 mM). Profiles are representative of three independent replicates. Inset shows the inflection temperatures (Ti) for each sample, average of three individual replicates ± SD. (C) Sedimentation profiles of *His*-DIPP1 mutants (R41A, top; N112S, middle; R41A/N112A, bottom) in the absence and presence of 2 mM IP_6_. The insets show the full sedimentation range compared with that of *His*-DIPP1, both without IP_6_, highlighting the absence of higher-order species with all mutants, and the marked reduction of even the minimal association state, presumably the dimeric form, for the double mutant R41A/N112A. All experiments were conducted in 20 mM Tris/HCl pH 7.5, 0.15 mM DTT (except in thermal stability experiments, 1 mM DTT), supplemented with 150 mM NaCl in the specified stability data.

Our crystallographic results reflect a tendency of DIPP1 to occlude its active site through protein-protein interactions. Altogether, this prompted us to search for evidence of DIPP1 oligomerization in solution. AUC sedimentation profiles of *Hs*DIPP1 under low ionic strength revealed the polydisperse nature of the protein under these experimental conditions, reflected in the presence of a number of oligomerization states with two major peaks at ca. 1.4 S and 1.9 S compatible with monomeric and dimeric forms, and higher order species sedimenting with larger *s*-values (Fig. 6A). Importantly, polydispersity of wt-DIPP1 is largely reduced in the presence of IP_6_ under these conditions, the profiles showing a single major peak with an *s*-value comparable to that of the alleged monomer observed in free protein (Fig. 6A). This is consistent with the mutual incompatibility of ligand binding and oligomerization as observed in the crystallographic structures (Fig. 5C). DLS experiments conducted under similar conditions rendered apparent translational diffusion coefficients (*D*_*app*_) in agreement with this behavior. Thus, wt*-*DIPP1 samples showed diffusion profiles corresponding to an ensemble of highly polydisperse species, with *D*_app_ values indicative of slow diffusion (higher order species; Figs. 6A and S8A). The presence of IP_6_ shifted the curves toward faster diffusion times, with a substantial increase in the apparent diffusion value (Figs. 6A and S8A). The diffusion value obtained for the main, discrete species in the latter profiles by a two species analysis (Fig. S8A) was (7.8 ± 0.8) 10^-7^ cm^2^/s. The combination of this value through the Svedberg equation with the normalized sedimentation value for the slower sedimenting species (*s*_20,w_ = 2.1 S), rendered a mass of ca. 23 kDa compatible with the monomer. The enzyme association trend is lost at higher ionic strength (i.e. 150 mM NaCl), with the sedimentation profiles showing a single peak compatible with the monomer disregarding the presence of IP_6_ (Fig. S8B) and a similar shift in the DLS profiles towards faster diffusion species (S8C,D). Interestingly, the effect of IP_6_ on the association state of DIPP1 is maintained at lower, close to physiological, concentrations of the ligand (Fig. S8C,D). Collectively, these results are coherent with the electrostatic character of interactions observed in the association interface between consecutive monomers. We observed that the thermal stability of wt-DIPP1 is more than 5 °C lower in absence of salt, (ca. 50 ºC; Fig. 6B). Nevertheless, the shape of the thermal stability profiles, with a steep signal change occurring in a small temperature interval, in indicative of properly folded proteins able to bind IP_6_ which produce a shift in the denaturation Ti of ca. 30 °C and 10 °C in the absence and presence of salt, respectively (Fig. 6B).

Next, we analyzed the effect of selected DIPP1 mutations at residues located at the protein-protein interfaces observed in the two crystallographic associations reported here (Figs. 5A and 5B). Specifically, we explored mutations at Arg41 (R41A) and Asn112 (N112S), both of which are essential for wt-DIPP1 dimer association (Fig. 5A). Whereas Arg41 also participates directly in the DIPP1-E108N fiber-like association (Fig. 5B), the role of Asn112 in this assembly cannot be excluded due to its key position in loop L3, whose conformation changes markedly in the fiber-like association (Fig. 5C, bottom-left). Under low ionic strength, AUC sedimentation profiles obtained for these mutants are consistent with species that are less prone to associate into larger oligomers, with considerably less polydisperse profiles (Fig. 6C). However, both single mutants retain the ability to mostly populate two association states, with peaks at ca. 1.3 S and 1.8 S for R41A; and 1.4 S and 2.0 S for N112S, compatible with monomeric and dimeric forms. By contrast, a double mutated DIPP1 sample (R41A/N112A) yielded a sedimentation profile with a major dominant peak at ca. 1.4 S, consistent with the monomeric form of the protein. As with wt-DIPP1, the sedimentation profiles in the presence of IP_6_ showed a single peak indicative of dimer dissociation upon ligand binding with these three mutants. Altogether, these results suggest that altering residues involved in the crystallographic DIPP1 protein-protein interfaces reduces higher-order associations as well as the dimer formation.

Interestingly, NMR experiments show that the DIPP1 orthologue *Sc*DDP1 self-associates in solution. The comparison of the signal intensities of NMR spectra taken under nearly identical sample conditions for wt-*Sc*DDP1 and a loop-deletion mutant (30 residues, named as *nose*), shows a dramatically lower intensity for wild type that can only be attributable as being wild type in oligomeric forms (Fig. S9). Furthermore, the NMR signals of non-exchangeable CH_3_ show a temperature dependence in the wt and not in the mutant, which indicates that wt-*Sc*DDP1 is involved in chemical processes sensitive to temperature (like oligomerization). This effect disappears after “nose” removal (Fig. S9B). Finally, the behavior of the NMR signals provides information about ligand binding. The three tryptophans, whose Hε1 resonances were tentatively assigned, are too far away to be significantly perturbed by ligand binding. Unexpectedly, “nose”-belonging Trp107 experiences large changes and is found in two forms upon titration with sub-stoichiometric IP_6_ concentration (Fig. S9C). Such behavior could be explained as an interference effect of ligand binding with nose-mediated self-association, which is in line with X-ray and hydrodynamic data obtained for *Hs*DIPP1.

In conclusion, our experimental data show that the DIPP1 family tends to associate with the concurrent occlusion of the active site, opening a window to propose a regulatory mechanism for this family mediated by protein self-association into species still able to respond to fluctuations in ligand concentration.

## Discussion

In this work, we found experimental evidence for three distinct IP_8_ binding modes in the human DIPP1 (*Hs*DIPP1) catalytic site: 1PP-SP, 1PP-TB, and 5PP. Among them, the 5PP (chair conformation) and unprecedented 1PP-TB (twist-boat conformation) modes appear fully productive for catalysis, correctly positioning the scissile phosphate for hydrolysis, at positions 5 and 1, respectively.

We have observed that PP hydrolysis at position 5 proceeds as expected, i.e. binding IP_8_ in a “canonical” chair conformation with substituents arranged one in an axial orientation and five in equatorial orientations (1ax/5eq). By contrast, PP hydrolysis at position 1 appears possible only when IP_8_ adopts a twist-boat conformation. In the case of 1-PP hydrolysis, the chair form of IP_8_, as described for 1-IP_7_, would produce severe steric clashes caused by the additional phosphate at position 5, mainly with L1 residues Ser39 and Arg41, and the subsequent Ile47. A major conformational rearrangement in the enzyme to accommodate this chair form seems unlikely, as comparison of multiple structural forms reveals no such changes. Computational docking, conformational energy analyses and molecular dynamics simulations also support the feasibility of the twist-boat conformation, despite it being energetically less favorable (~ 5 kJ/mol in solution) than the chair form. Notably, ring-flipped (5ax/1eq) conformations of IP_8_ have previously been observed in solution [17], echoing adoption by IP_6_ of the 5ax/1eq chair conformation at high pH [40]. This energetic landscape highlights the flexibility of IP_8_ and its ability to adapt to the highly basic and spatially constrained environment of the DIPP1 active site. Importantly, protein contributions and metal ions could alter the equilibrium between inositide conformations.

Therefore, our data provide experimental evidence for both 1-PP and 5-PP hydrolysis of IP_8_. Previous studies suggest that DIPP1 hydrolyzes the 5-PP position in IP_8_ more efficiently than the 1-PP [2], and that it preferentially hydrolyzes 1-IP_7_ over 5-IP_7_ [2,14]. Here, we also show that when a mixture of 1- and 5-IP_7_ is present, 1-IP_7_ is the preferred substrate. Phosphate release assays confirm that 1-IP_7_ is a better substrate than 5-IP_7_ or IP_8_ (Figs. 4B and S7). Unfortunately, the absence of IP_7_ intermediates precluded determination of the preferred IP_8_ cleavage site. However, like others [2,14], our experiments suggest that the conversion of IP_8_ to IP_7_ is much slower than IP_7_ hydrolysis itself. It is possible that the pH- and Mg^2+^-dependent solution equilibrium of 1ax/5eq and 5ax/1eq (chair) conformations might limit overall kinetic velocity for IP_8_ vs 1-IP_7_, since the latter has lower tendency at any given pH/Mg^2+^ condition to adopt the 5ax/1eq chair conformation [17]. The significance of the crystallographic twist-boat conformation to solution phenomena is more difficult to rationalize; that it represents a transitional intermediate in 1ax/5eq to 5ax/1eq conversion is an intriguing possibility.

Speculation aside, and in the absence of definitive experimental data revealing the preferred IP_8_ cleavage site, the fact that crystallography captured a catalytically competent form of an IP_8_ analogue in a twist-boat conformation for 1-PP hydrolysis (1PP-TB binding mode) suggests that hydrolysis at this position is relevant. It is possible that certain conditions favor the capture of this less stable conformation in crystals, despite it not having been observed in solution. Indeed, crystals obtained with the R89S mutant favor twist-boat (1PP-TB mode) capture. This may be related to the proximity of Arg89 to the pyrophosphate at the non-hydrolyzing position (Fig. 1B). Similarly, crystallization conditions and the use of a non-hydrolysable IP_8_ analogue (bearing a P-C-P bridge), may stabilize specific binding modes. In any case, the binding energy to DIPP1 of IP_8_ in the 1PP-TB mode may make this mode accessible for catalysis, with the extended crystallization times favoring the stabilization of rare but energetically accessible conformations.

Nevertheless, it should be noted that the most frequently observed binding mode in our crystals is the semi-productive 1PP-SP mode, with IP_8_ in a canonical chair conformation. This mode has been also captured in the *Saccharomyces cerevisiae* orthologue *Sc*DDP1 [16]. This third binding mode may itself represent a physiological IP_8_ interaction with DIPP1, possibly involved in enzyme inhibition under substrate excess conditions.

To conclude here, it seems reasonable to propose that DIPP1 specificity and catalytic efficiency depend on the cellular milieu of competing PP-InsPs pools, impacted by prevailing pH and Mg^2+^. For example, 1-IP_7_ is known to be less abundant than 5-IP_7_ in cells [2,3], the reported intracellular concentrations for highly phosphorylated InsPs being approximately 10-100 µM for IP_6_, ~0.05 µM for 1-IP_7_, ~0.5-5 µM for 5-IP_7_ and ~0.1-0.5 µM for IP_8_ [41–44]

In parallel, it is expected that this family of enzymes is under strict control and regulation. Members have an extremely basic active site that can act as an attractor of proteins or other metabolites, as has been reported for some kinases [45]. In addition, their regulation is particularly relevant in the context of cellular stress responses, where enzymes like DIPP1 might adapt their activity to fluctuating intracellular conditions. Our data suggest that DIPP1 oligomerization could represent an additional regulatory mechanism for modulating its activity. Oligomer formation may transiently occlude the active site, acting as an autoregulatory strategy in situations of substrate needs, product excess or fluctuations in physiological conditions, including salt and phosphate levels. This hypothesis is supported by our structural, as well as in solution (AUC and DLS) and mutagenetic, experimental data that reveal a monomer-oligomers equilibrium influenced by salt concentration and ligand binding. However, our data suggest that this equilibrium occurs transiently and under specific conditions. There are previous studies showing that oligomerization is a regulatory strategy found in some enzymes with highly basic active sites [46]. Interestingly, we observed phosphate ions at both dimer and oligomer interfaces proposed, which may reflect a Pi function in this equilibrium. Given the well-established link between PP-InsPs and cellular phosphate sensing and regulation [47–50], it is plausible that Pi itself may participate in feedback mechanisms controlling PP-InsP levels. The action of these enzymes to the regulation of polyPs further link their activity to phosphate homeostasis [10]. Besides, we must also consider that members of this family might undergo phosphorylation *in vivo*, even by their own substrates, which could further regulate protein oligomerization or other events. Interestingly, the serine residues in L1 (Ser38 and Ser40) are predicted phosphorylation sites and are spatially close to Arg41, which is involved in Pi recognition at the crystallographic dimer interface presented in this work. We think that this regulation could be kingdom specific as, for example, *Sc*DDP1 shows a distinct insertion that seems to be involved in auto-association events as we observed herein by NMR experiments. Alternatively, our data might reflect a trend of the DIPP1 family to make heterotypic protein-protein interactions.

Though merely speculative at this point, further possible implications of DIPP1 family oligomerization may involve arrangement into dynamic larger assemblies as biomolecular condensates driven by reversible weak interactions [51,52], structures implicated in physiological and pathological processes [53]. Condensates have been found, among others, within kinases involved in cell signaling functions such as those mediated by the Hippo, the NF-κB and the JAK/STAT signaling pathways [54]. There is growing evidence that clusters of basic residues can promote phase separation phenomena leading to the formation of condensates that are highly dependent on the external environment [52]. It is tempting, considering the highly basic surface of DIPP1 and its ability to self-associate into transient, reversible arrangements responsive to ligands and experimental conditions, to speculate about the possibility of DIPP1 family members forming biomolecular condensates *in vivo*. Exploring this avenue, and any other physiologically relevant role of DIPP1 association, require thorough in-depth studies, and using conventional biochemical and biophysical approaches alone may prove challenging. Instead, progress is more likely to come from orthogonal strategies also including cell-based approaches such as co-immunoprecipitation, FRET/BRET, live-cell fluorescence imaging, cross-linking or proximity labelling, complemented by macromolecular crowding *in vitro* assays, which could provide new insights into the spatial and temporal regulation of inositol pyrophosphate metabolism.

## Conclusions

Our findings significantly advance the understanding of DIPP1 function and regulation, emphasizing the critical role of ligand flexibility in accommodating IP_8_ and highlighting oligomerization as a potential regulatory mechanism. These insights contribute to elucidating the broader implications of DIPP1 in inositol pyrophosphate metabolism, cellular phosphate homeostasis, and stress response pathways.

While certain limitations of the study must be considered, including the use of non-hydrolyzable IP_8_ analogues and the possibility of non-physiological oligomeric assemblies captured in crystals, both computational analyses and complementary experimental data observed in solution support our conclusions. Future studies employing cell-based methods and biophysical approaches will be important to assess the physiological relevance of the observed IP_8_ conformations and oligomerization behavior reported here, and to test whether DIPP1 undergoes environment-sensitive assembly or condensation in cells.

## Supplementary Material

Supplementary Information

Supplementary Legends

Supplementary Video 1

Supplementary Video 2

Supplementary Video 3

## Data Availability

All the atomic coordinates and the structure factors of the crystal structures are deposited in the Protein Data Bank (https://www.rcsb.org) under the submission codes 9T49 and 9T4A (DIPP1/PCP-IP_8_ in 1PP-SP mode); 9T48 (DIPP1-R89S/PCP-IP_8_ in 1PP-TB and 1PP-SP modes); 9T4B (DIPP1-R89S/PCP-IP_8_ in 5PP mode); 9T4C (DIPP1-H91E/PCP-IP_8_ in 1PP-SP and out-mode); 9T4D (DIPP1-R41A/5-IP_7_); 9T4G (DIPP1/IP_6_); 9T4H (DIPP1-R41A/IP_6_); 9T4I (DIPP1-R89S/IP_6_); 9T4J (DIPP1-H91E/IP_6_); 9T4K (DIPP1-H91M/IP_6_); 9T4L (DIPP1-E108N/IP_6_); 9T4M (DIPP1-N112S/IP_6_); 9T4E (DIPP1); 9T4F (DIPP1-E108N).
