# Supplementary material for: The DIPP1 family binds IP8 in catalytically-productive twist-boat and chair conformations and associates in a ligand-dependent manner
Source: Int J Biol Macromol. Author manuscript; Available in PMC 2026 Aug 1. (PMC7619153; doi:10.1016/j.ijbiomac.2026.152715)
Supplement: Supplementary Information [file EMS214218-supplement-Supplementary_Information.pdf]

Supplementary data

The DIPP1 family binds IP_8 in catalytically-productive twist-boat and chair conformations and associates in a ligand-dependent manner

David Casas-Florez[1], Hayley Whitfield[2], Jose M. Pérez-Cañadillas[3], Begoña Monterroso[1], Andrew M. Riley[4], María A. Márquez-Moñino[1,5], Megan L. Shipton[4], Julia Sanz-Aparicio[1], Charles A. Brearley[2], Barry V.L. Potter[4] and Beatriz González[1]*

[1]Department of Crystallography and Structural Biology, Institute of Physical-Chemistry Blas Cabrera, CSIC, Serrano 119, 28006 Madrid, Spain.
[2]School of Biological Sciences, University of East Anglia, Norwich Research Park, Norwich NR4 7TJ, UK.
[3]Department of Biological Physical Chemistry, Institute of Physical-Chemistry Blas Cabrera, CSIC, Serrano 119, 28006 Madrid, Spain.
[4]Drug Discovery & Medicinal Chemistry, Department of Pharmacology, University of Oxford Mansfield Road, Oxford, OX1 3QT, UK.
[5]Present address: Structural Glycoimmunology Laboratory, Biobizkaia Health Research Institute, 48903 Barakaldo, Spain

* Correspondence: xbeatriz@iqf.csic.es (B.G.)

Supplementary materials and methods

Enzyme assays

Analysis of reaction products by HPLC and detection with ferric ion

DIPP reaction products were resolved by HPLC on a CarboPac PA-200 column according to Whitfield *et al.* [1] Reactions of 10 μL containing 40 ng or 80 ng wt- or mutant DIPP1 enzyme with 100 μM substrate in 20 mM HEPES pH 7.2, 100 mM KCl, 0.8 mM $MgCl_2$, 20 μM Na_2EDTA were incubated at 37 °C for 15 minutes. Reactions were stopped by addition of 30 μL 20 mM Na_2EDTA, 100 mM NaF, pH 10. For assays with mixed equimolar or individual 1-IP_7 and 5-IP_7 and IP_8 substrates, 80 ng enzyme was used.

Phosphate release assay

Reactions of 50 μL containing 400 ng wt-DIPP1 enzyme with 200 μM substrate were incubated at either pH 7 or pH 8.2 and either 200 μM $MgCl_2$ or 1 mM $MgCl_2$ in conditions otherwise described above. From these reactions, 10 μL aliquots were removed and the reaction stopped with 30 μL 20 mM Na_2EDTA, 100 mM NaF, pH 10, for analysis by suppressed ion-conductivity HPLC. Separately, in a 384-well plate, 10 μL enzyme reaction was mixed with 10 μL colour reagent in triplicates (4 parts 1.5% w/v ammonium molybdate in a 5.5% v/v sulphuric acid solution; 1 part 10.8% w/v iron(II) sulphate solution). After 15 minutes incubation at room temperature, the absorbance at 700 nm was measured using a Hidex Sense (LabLogic Systems, UK) microplate reader. Measurements of substrate background absorbance were subtracted from the assay absorbance values and the resulting values converted to phosphate using a standard curve of 1-5000 μM KH_2PO_4.

Suppressed ion-conductivity HPLC

Aliquots of products from phosphate release assays were analyzed by suppressed ion-conductivity on a Dionex (UK) ICS-2100 system after resolution on a 250x2 mm AS11 (Dionex) column with 50x2 mm AG11 (Dionex) guard column. The column, held at 30°C, was eluted at a flow rate of 0.35 mL min^{-1} with a gradient: time (min), KOH (mM); 0,5; 80, 80; delivered with gradient function '5' in the Chromeleon v.6 software (Dionex, UK). The anion suppressor current was set at 99 mA. The column was washed with 5 mM KOH for 10 minutes between injections. Aliquots of 12 μL were injected. Chromatographic *x*, *y* data was exported as .csv files and replotted in GraphPad Prism v.6.

Light scattering

Inositol hexakisphosphate, 200 μM in 20 mM HEPES pH 7 or 8.2, 100 mM KCl, 20 μM Na_2EDTA, was supplemented with 0, 1 or 5 mol equivalent (0, 200 μM or 1 mM) $MgCl_2$

and incubated at 37 ºC for 15 min. Aliquots of 0.15 mL were immediately transferred to a 4 x 4 mm pathlength quartz glass cuvette (volume 0.2 mL) placed in a Jasco FP8-500 Spectrofluorimeter, held at 37 ºC. The sample was excited at 280 nm with 5 nm bandpass and light at 90º passing the emission monochromator was scanned from 500 – 600 nm with 5 nm bandpass. For pure solvent, the peak observed at ca. 560 nm represents second-order Rayleigh scattering of excitation light by the emission monochromator diffraction grating. Light is also scattered by colloidal particles and so scattering of excitation light allows detection of precipitates. Spectroscopic *x*, *y* data was exported as .csv files and replotted in GraphPad Prism v.6.

IP6K2 expression and purification

*Hs*IP6K2 was cloned in pKLSL-vector and expressed in *E. coli* BL21 Star. The cells were grown in 2TY medium supplemented with 50 µg/mL kanamycin at 37 °C until an OD_{600} = 0.9. Protein overexpression was induced with 0.4 mM IPTG and incubating for 16 hours at 16 °C. Cells were resuspended in buffer F (20 mM Tris/HCl pH 8 [4 °C] and 200 mM NaCl) and sonicated. Clarified cell lysate was loaded onto a home-prepared Sepharose CL4 column equilibrated in buffer F. Protein elution was performed with buffer F plus 10 mM lactose, and after 1:3 dilution in buffer G (20 mM Tris/HCl pH 8, 1 mM DTT) it was loaded onto a 5 mL HiTrap™ Heparin HP column (Cytiva) pre-equilibrated with buffer H (20 mM Tris/HCl pH 8 [4 °C], 50 mM NaCl, 1 mM DTT). Elution was performed with a salt gradient of 0.05–1 M NaCl in 100 mL and LSL-tag was removed as described in the main manuscript. Subsequently, the sample was loaded onto a 1 mL home-prepared Sepharose CL4 equilibrated in buffer F and the flow-throw was selected. Finally, the protein sample was passed through a 16/600 Superdex200 column (GE Healthcare) equilibrated with buffer I (20 mM Tris/HCl pH 8 [4 °C], 150 mM NaCl, 1 mM DTT) supplemented with 2 mM IP_6 and 1 mM $MgCl_2$. The sample was concentrated to approximately 2 mg/mL and stored at -80 °C until use.

5-PP-IP_7 biosynthesis and identification

Based on the Puschmann *et al.* protocol [2], we prepared a 600 mL reaction mixture consisting of 250 µM IP_6 (Sigma-Aldrich), 2 mM ATP, 7 mM $MgCl_2$, 5 mM creatine phosphate, 20 mM NaCl, 20 mM MES pH 6.4, 1 mM DTT, and water. This mixture was adjusted to pH 6.4 and preincubated for 10 minutes at 37 °C. Subsequently, 0.3 µM LSL-*Hs*IP6K2 and 1 U/mL creatine kinase were added, mixed thoroughly, and allowed to react, without agitation, for 30 minutes. Following steps are similar to those described [2]. From 50 g of IP_6 we obtained 30 g of lyophilized solid.

The conversion of IP_6 to solid 5-IP_7 was further analyzed by NMR (see next section) and crystallography. Following other authors' approach for product identification [3], we produced and analyzed crystals of DIPP1 in the presence of 10 mM of the lyophilized product (26-30% PEG 6000, 0.1 M sodium acetate pH 6, 0.2 M LiCl, 10 mM $MgCl_2$, +/- 10 mM NaF). Crystallization, cryoprotection, data collection and structural determination were performed as stated in the main text. Electron density maps for crystals grown in the presence of NaF were compatible with the inclusion of 5-IP_7 (Fig. S10).

NMR experiments

The enzymatic conversion of IP_6 to IP_7 by *Hs*IP6K2 (Fig. S10) was monitored by ^{13}P NMR in a 600 MHz Bruker AV600 spectrometer equipped with a ^{31}P cooled channel. For this, we prepared a biosynthesis mixture scaled up to a final volume of 500 µL, increasing the IP_6 concentration to 0.5 mM (see above). Samples were quenched at initial point and after 30 min by lowering the pH to 1.6 (by adding 0.2 M glycine, 35% HCl) and the ^{31}P NMR spectra recorded (512 scans with ^{1}H decoupling).

The behavior of *Sc*DDP1 in solution was analyzed by NMR. Reference ^{1}H NMR spectra (1000 scans) were recorded for wild-type *Sc*DDP1 and Δnose-*Sc*DDP1 mutant at 100 µM protein concentration, in 150 mM NaCl and 0.1 mM DTT, at pH 6.7 buffer conditions (with 10% D_2O) and at 298 K (25 ºC), in an 800 MHz Bruker AV800 spectrometer equipped with a cryoprobe. Additional ^{1}H NMR spectra for wild-type and mutant DDP1 (100 µM) were recorded at two IP_6 titration points with 100 µM and 600 µM ligand concentrations under the same temperature and buffer conditions. For temperature series, shorter datasets (256 scans) of both proteins were recorded at 293, 298, 303, 308 and 318 K.

All the NMR experiments were processed with Topspi4.3 (Bruker) and MestreNova

Supplementary references

1. H. Whitfield *et al.* An ATP-responsive metabolic cassette comprised of inositol tris/tetrakisphosphate kinase 1 (ITPK1) and inositol pentakisphosphate 2-kinase (IPK1) buffers diphosphosphoinositol phosphate levels, *Biochem. J.* 477 (2020) 2621–2638.
2. R. Puschmann, R.K. Harmel & D. Fiedler. Scalable Chemoenzymatic Synthesis of Inositol Pyrophosphates, *Biochem.* 58 (2019) 3927–3932.
3. G. Zong, S.B. Shears, & H. Wang, Structural and catalytic analyses of the $InsP_6$ kinase activities of higher plant ITPKs, *The FASEB J.* 36 (2022).

Table S1. Crystal data processing and refinement statistics. (Table in the excel file)

Table S2. RDKIT IP_8 conformers**.**

Conformer Number	Energy (kJ/mol)	Torsions	Conformation
**40**	37.92	[56.93, -56.75, 56.24, -56.35, 56.83, -56.91]	Chair 1
**37**	38.34	[56.73, -56.52, 56.65, -57.22, 57.57, -57.23]	Chair 1
**27**	39.23	[56.32, -56.76, 56.61, -56.17, 56.01, -56.0]	Chair 1
**35**	39.42	[56.78, -56.46, 55.82, -55.74, 56.51, -56.89]	Chair 1
**16**	40.21	[56.12, -56.84, 57.08, -56.75, 56.27, -55.88]	Chair 1
**13**	40.35	[57.66, -56.86, 55.43, -55.28, 56.4, -57.41]	Chair 1
**7**	40.41	[56.87, -56.71, 56.15, -55.99, 56.63, -56.93]	Chair 1
**6**	40.44	[58.02, -58.08, 56.62, -55.56, 55.59, -56.69]	Chair 1
**49**	40.48	[56.41, -56.49, 56.57, -56.74, 56.87, -56.58]	Chair 1
**33**	40.55	[56.95, -56.74, 56.17, -56.11, 56.52, -56.79]	Chair 1
**21**	42.02	[-54.83, 53.82, -53.45, 53.57, -54.1, 54.95]	Chair 2
**32**	42.09	[-55.06, 53.7, -53.5, 54.12, -54.85, 55.61]	Chair 2
**29**	42.28	[55.43, -54.75, 55.15, -57.06, 58.23, -57.12]	Chair 1
**41**	42.3	[-55.83, 55.34, -54.14, 53.1, -53.32, 54.81]	Chair 2
**4**	42.58	[-55.05, 54.92, -54.43, 53.58, -53.28, 54.18]	Chair 2
**45**	42.61	[-55.49, 55.49, -54.61, 53.44, -53.24, 54.43]	Chair 2
**18**	42.83	[-54.49, 53.37, -53.59, 54.3, -54.72, 55.08]	Chair 2
**2**	43.16	[-55.23, 54.47, -53.75, 53.53, -53.95, 54.94]	Chair 2
**31**	43.64	[-54.84, 54.35, -53.64, 53.28, -53.77, 54.6]	Chair 2
**46**	43.64	[-55.72, 55.57, -54.37, 53.06, -53.1, 54.56]	Chair 2
**15**	43.84	[-55.43, 54.51, -53.87, 53.6, -53.99, 55.06]	Chair 2
**10**	44.06	[-54.61, 52.04, -51.84, 53.76, -55.94, 56.52]	Chair 2
**5**	44.38	[-54.87, 55.01, -54.86, 54.25, -53.68, 54.1]	Chair 2
**47**	44.92	[-54.99, 54.73, -54.64, 54.59, -54.76, 55.19]	Chair 2
**20**	46.15	[-56.35, 54.04, -52.73, 53.66, -55.36, 56.84]	Chair 2
**22**	50.02	[-64.31, 30.91, 29.67, -61.22, 28.1, 33.05]	Twist-Boat 1
**38**	50.39	[-62.54, 28.64, 31.64, -62.1, 28.36, 32.23]	Twist-Boat 1
**42**	50.46	[28.95, -62.16, 31.53, 28.74, -61.85, 31.37]	Twist-Boat 2
**14**	51.44	[-62.56, 28.92, 31.97, -63.07, 29.41, 31.5]	Twist-Boat 1
**9**	51.72	[30.02, -63.31, 32.72, 27.55, -60.72, 30.24]	Twist-Boat 1
**8**	51.85	[30.46, -62.0, 29.69, 30.69, -62.02, 29.73]	Twist-Boat 2
**1**	52.02	[32.2, -63.02, 29.6, 30.93, -61.48, 28.22]	Twist-Boat 2
**28**	52.09	[-30.25, -30.83, 63.11, -30.59, -30.19, 62.48]	Twist-Boat 3 ~ experimental
**19**	52.14	[-31.93, -28.88, 61.17, -28.92, -31.55, 63.89]	Twist-Boat 3 ~ experimental
**11**	52.27	[30.01, -61.83, 29.83, 30.35, -62.05, 30.16]	Twist-Boat 2
**17**	52.32	[26.34, 32.76, -61.43, 27.66, 31.65, -60.29]	Twist-Boat 4
**26**	52.38	[60.68, -30.99, -28.71, 61.72, -31.88, -27.39]	Twist-Boat 5
**30**	52.43	[31.96, -60.69, 26.41, 33.15, -61.87, 27.85]	Twist-Boat 2
**39**	52.46	[-30.21, -31.3, 65.59, -34.49, -26.61, 60.93]	Twist-Boat 3 ~ experimental
**25**	52.61	[-31.13, 62.67, -30.43, -30.09, 61.79, -29.35]	Twist-Boat 6
**36**	52.69	[28.27, 31.01, -60.66, 27.52, 32.5, -61.91]	Twist-Boat 4
**24**	52.86	[29.06, 30.83, -60.55, 27.17, 32.95, -62.77]	Twist-Boat 4
**3**	53.33	[61.64, -33.27, -26.33, 61.07, -32.78, -26.97]	Twist-Boat 5
**12**	53.47	[29.94, -65.26, 35.89, 24.71, -59.93, 30.98]	Twist-Boat 2
**23**	53.57	[-31.41, -29.89, 64.4, -33.8, -27.05, 61.66]	Twist-Boat 3 ~ experimental
**0**	53.73	[-61.69, 29.84, 31.02, -64.16, 32.34, 28.8]	Twist-Boat 1
**34**	54.37	[63.02, -33.6, -26.57, 60.37, -30.83, -29.05]	Twist-Boat 5
**48**	54.97	[-28.02, -32.52, 62.73, -28.6, -31.74, 62.04]	Twist-Boat 3~ experimental
**43**	55.05	[62.61, -33.55, -26.3, 59.83, -30.66, -28.78]	Twist-Boat 5
**44**	56.12	[-63.63, 28.87, 32.26, -62.67, 27.84, 33.34]	Twist-Boat 1

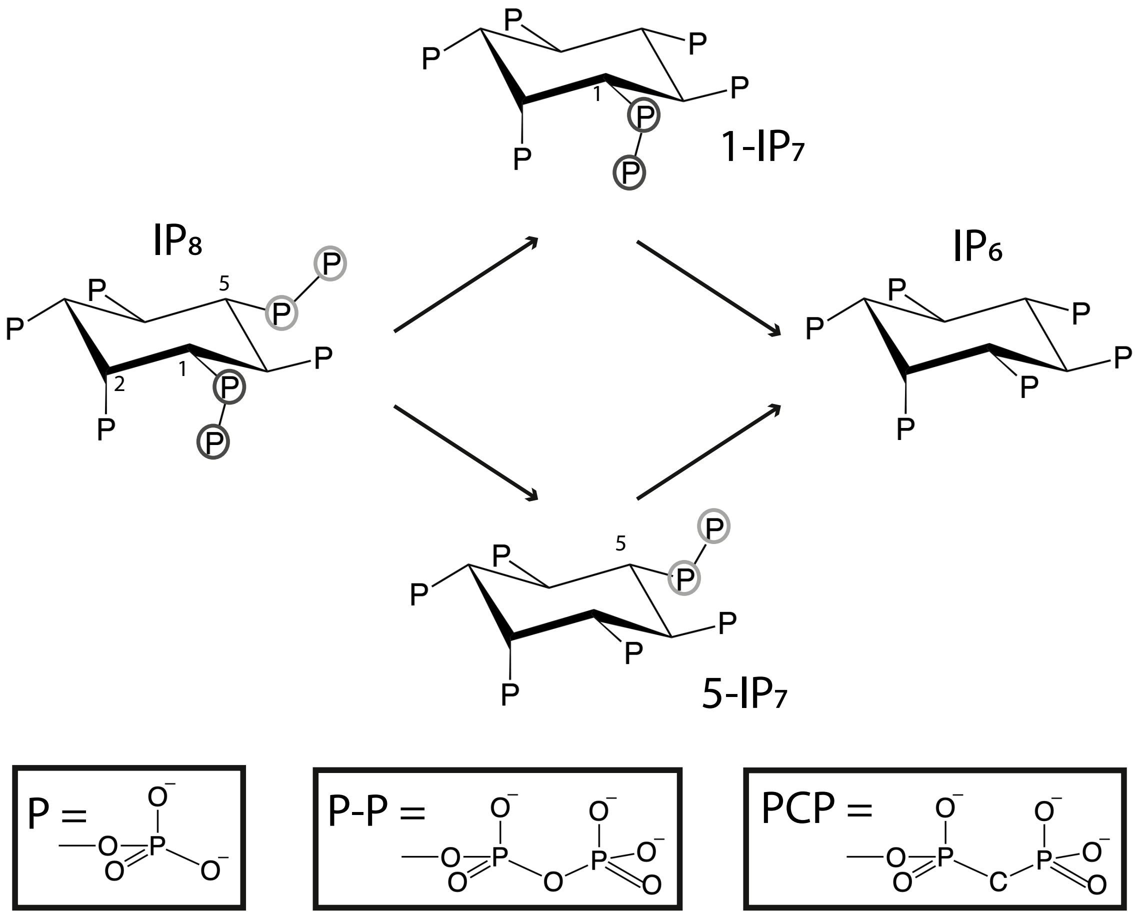

Fig. S1. Reaction catalyzed by DIPP1.

Schematic representation of the reaction catalyzed by DIPP1. The boxes below specify the more detailed nature of the abbreviations: P (phosphate), P-P (pyrophosphate) and PCP (non-hydrolyzable pyrophosphate).

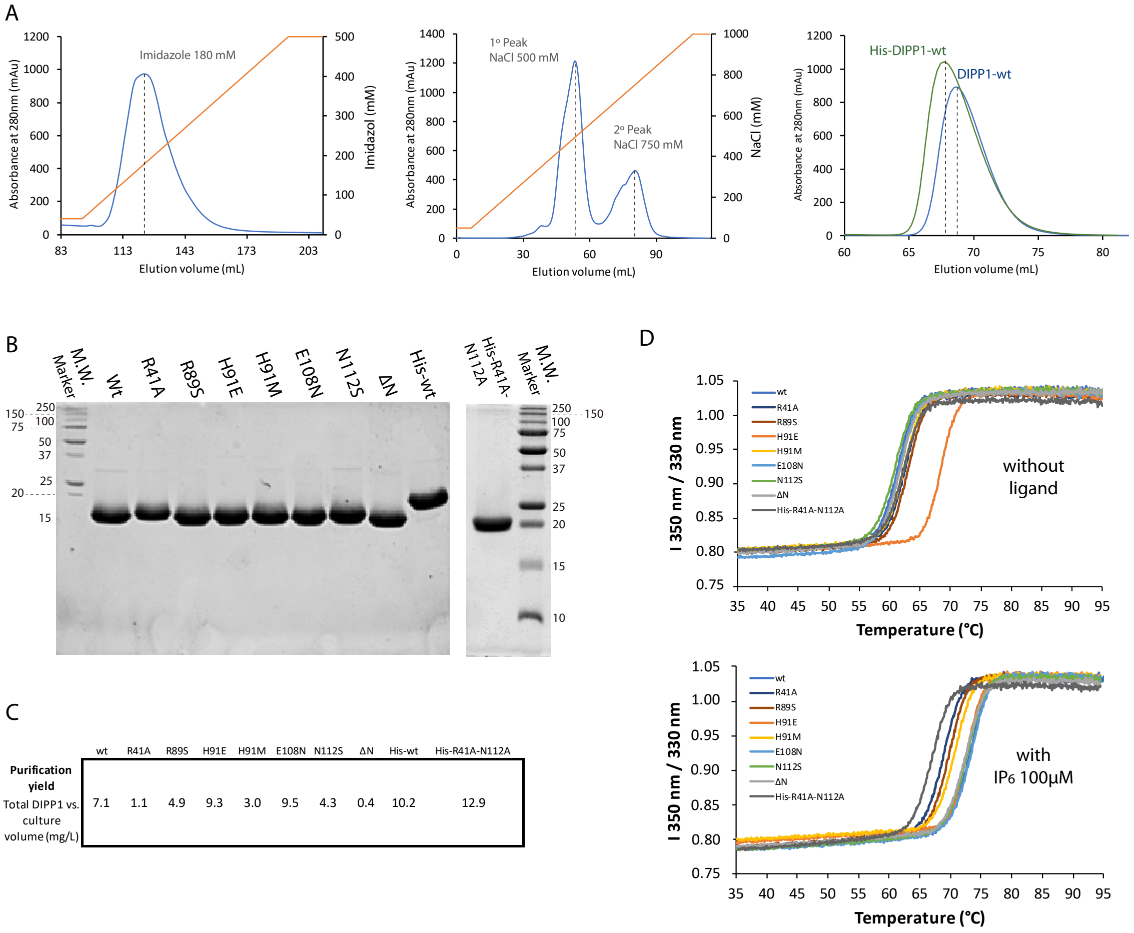

	wt	R41A	R89S	H91E	H91M	E108N	N112S	ΔN	His-wt	His-R41A-N112A
**Purification yield** Total DIPP1 vs. culture volume (mg/L)	7.1	1.1	4.9	9.3	3.0	9.5	4.3	0.4	10.2	12.9

Fig. S2. Biochemical and biophysical characterization of DIPP1 protein samples. (A) Representative chromatograms from wt-DIPP1 purification showing: (left) His-tag affinity chromatography with an imidazole gradient (mM); (middle) heparin affinity chromatography with a NaCl gradient (mM), the first peak eluting at ~500 mM NaCl was selected for the subsequent step; and (right) size-exclusion chromatography profiles of wt-DIPP1 before and after His-tag removal by TEV protease. (B) SDS–PAGE analysis of all DIPP1 variants studied in this work after size-exclusion chromatography and concentration for crystallography. Molecular weight markers are indicated for reference (values in kDa). (C) Summary of protein yields obtained during purification for all DIPP1 samples (per liter of culture, as indicated). (D) Thermal denaturation profiles for DIPP1 samples, in absence (top) and presence (bottom) of IP_6, measured as the intrinsic fluorescence 350/330 nm ratio.

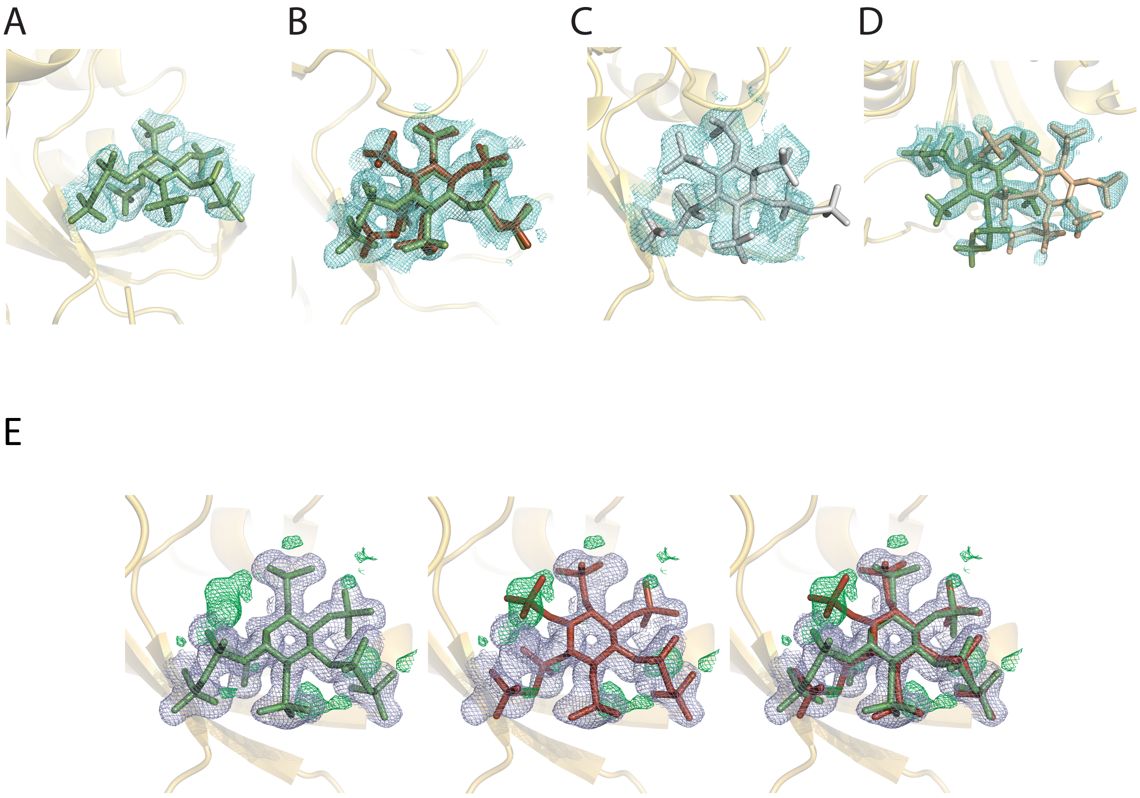

Fig. S3. Electron density 2Fo-Fc maps for DIPP1/PCP-IP$_8$ crystal complexes.
(A) Map for a wt-DIPP1 crystal complex showing PCP-IP$_8$ in the 1PP-SP mode (green). (B) Map for a DIPP1-R89S crystal complex showing PCP-IP$_8$ binding in two mixed modes: the 1PP-SP mode (green) and 1PP-TB mode (brown). (C) Map for a DIPP1-R89S crystal complex showing PCP-IP$_8$ in 5-PP mode (grey). (D) Map for a DIPP1-H91E crystal complex showing PCP-IP$_8$ in two mixed modes: 1PP-SP mode (green) plus an outside-the-catalytic-site mode (cream). The maps have been contoured at 1.3, 0.9, 1.3 and 1.0 sigma level respectively. (E) 2Fo-Fc map (blue, contoured at 1σ) and Fo-Fc map (green, positive peaks, contoured at 1σ) for wt-DIPP1 crystals refined with PCP-IP$_8$ in 1PP-SP mode (left). PCP-IP$_8$ in 1PP-TB mode (middle) and both PCP-IP$_8$ conformers (right) are superposed onto these maps to illustrate how TB conformation explains the positive peaks observed in the Fo-Fc map.

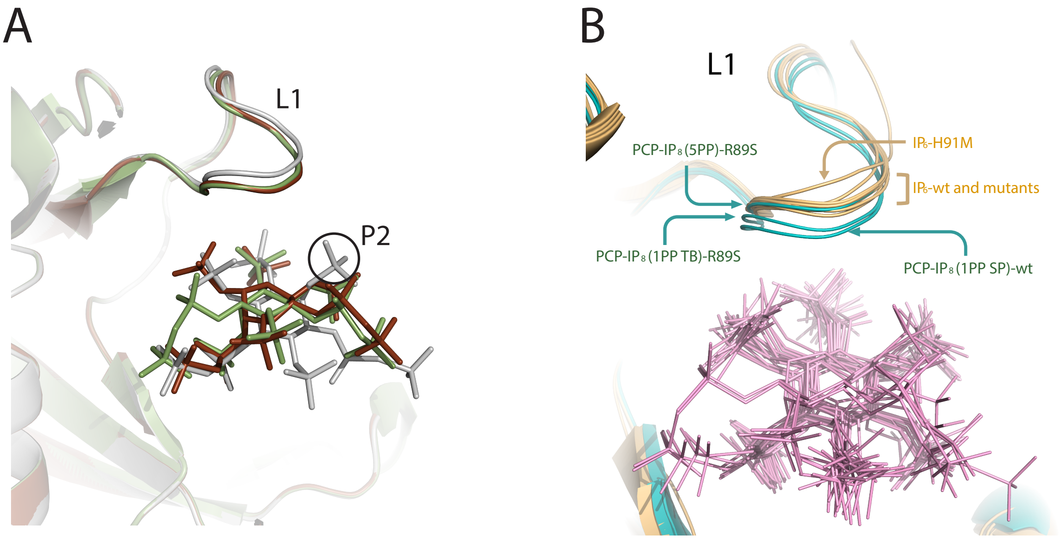

Fig. S4. Movement of Arg41 loop (L1).

(A) Superposition of DIPP1 structures obtained in the presence of PCP-IP_8 shows that the structure harboring the IP_8 analogue in the 5PP mode (white) adopts a more open L1 conformation than that observed in the 1PP modes (green, SP; brown, TB) to accommodate axial P2. (B) Superposition of all DIPP1 structures obtained in this work highlights the movement of loop L1. Structures harboring the IP_8 analogue in the 1PP mode show L1 positioned closer to the active site

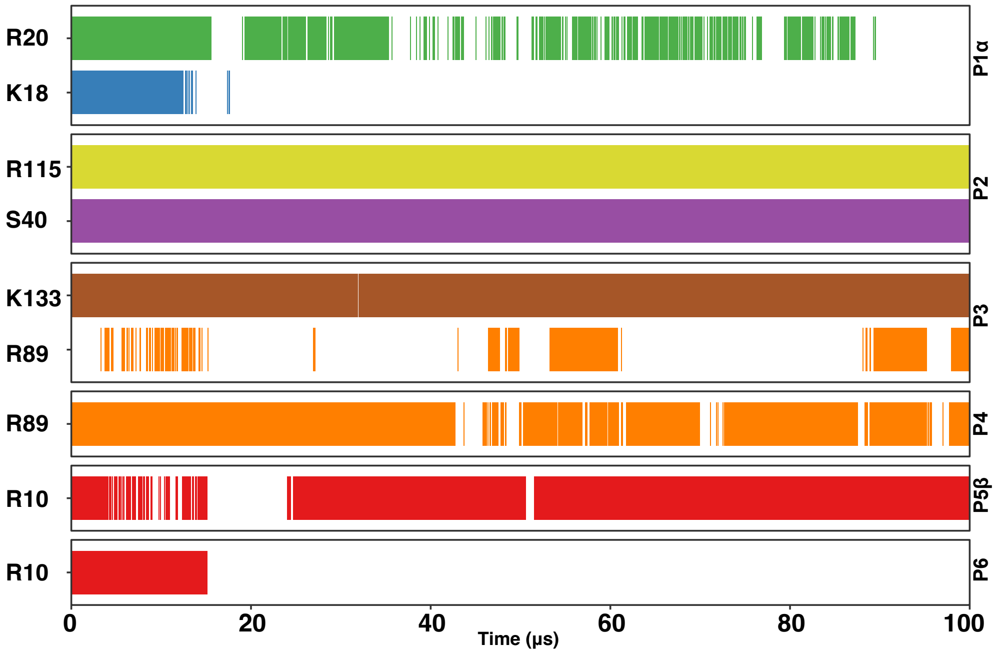

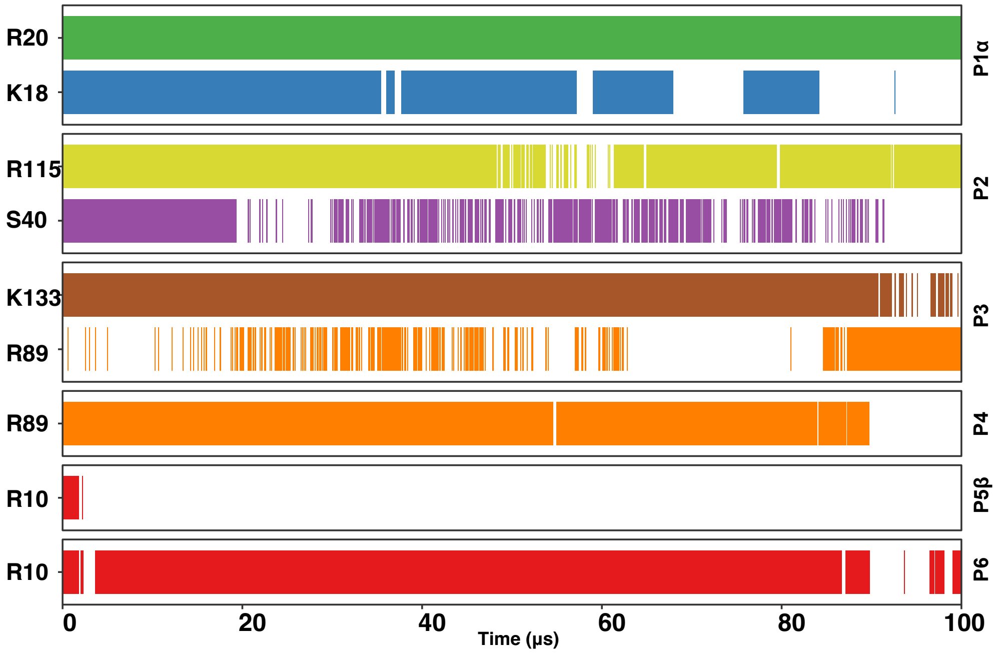

Fig. S5. Hydrogen bond events between IP_8 phosphates and DIPP1 selected residues. Representation of selected DIPP1 donor sidechains and their interacting IP_8 phosphate groups during two 100 μs MD production runs (the other production shown in Fig. 2C in the main text). Stick representations are color coded by protein residue. Notably, Arg89 and Arg10 form newly observed interactions: Arg89 was mutated to serine in the crystal structure, and Arg10 was engaged in crystal contacts.

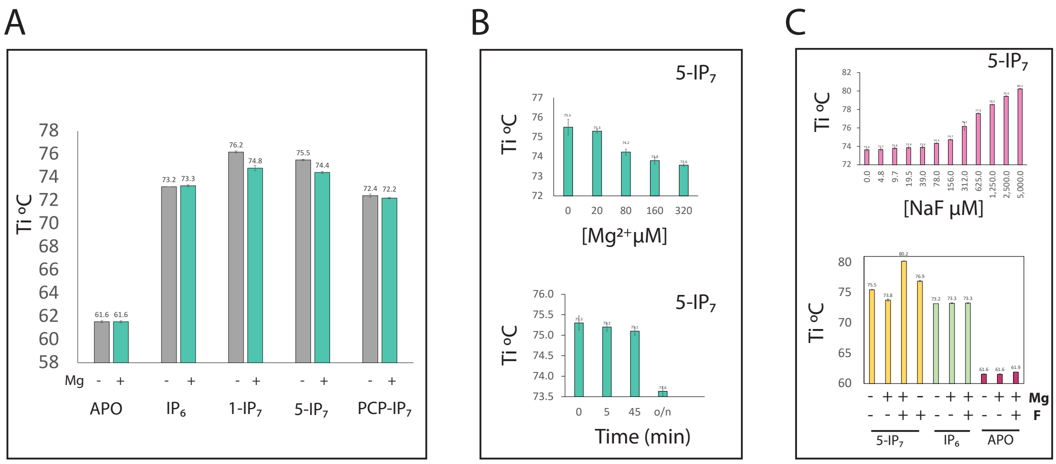

Fig. S6. Effect of Mg^{2+} on the stability of the wt-*Hs*DIPP1 complexes.

(A) Effect of Mg^{2+} on *Hs*DIPP1 inflection temperature (Ti), indicative of the thermal stability of the species in solution, in the absence and presence of different ligands. (B) Effect of Mg^{2+} concentration (top) and hydrolysis reaction time (bottom) on DIPP1/5-IP$_7$ thermal stability. It decreases when increasing Mg^{2+} concentration and hydrolysis reaction time, probably due to substrate processing rendering a complexes population enriched in DIPP1/IP$_6$. (C) Effect of NaF concentration on the stability of the DIPP1/5-IP$_7$ complex with Mg^{2+} (top) and thermal stability of *Hs*DIPP1 and its complexes with the substrate (5-IP$_7$) or the product (IP$_6$) with and without Mg^{2+} and/or 5 mM NaF (bottom). In all these experiments we used the biosynthesized 5-IP$_7$. DIPP1 concentration is 10 µM, inositide is 100 µM and, when present, Mg^{2+} is 80 µM (A), 5 µM (B, bottom) and 160 µM (C), or as stated. All samples were diluted in 20 mM Tris/HCl pH 7.5, 150 mM NaCl, 1 mM DTT (buffer E). Measurements were performed in triplicate, and the graphs show mean values with error bars representing standard deviation.

In summary: Mg^{2+} affects the thermal stability of *Hs*DIPP1, in a concentration-dependent manner, only when in complex with hydrolyzable substrates (1-IP$_7$ and 5-IP$_7$). At the conditions tested, stability of the samples with magnesium, lower than without the cation, shifted towards the values obtained for the complex of *Hs*DIPP1 with the product (IP$_6$). We can reasonably attribute this to an increase in IP$_7$ digestion triggered by Mg^{2+}, as with the non-hydrolyzable ligand 1-PCP-IP$_7$ (PCP-IP$_7$), as well as with IP$_6$, the stability variation is negligible. To check this, we carried out experiments to determine the dependence of DIPP1/5-IP$_7$ stability on increasing Mg^{2+} concentration and hydrolysis reaction times (B) that showed an evident decreasing trend in thermal stability. In agreement, the Ti of *Hs*DIPP1 in the presence of IP$_6$ (Fig.4A in the main manuscript) is similar to the Ti after processing 5-IP$_7$ (B, bottom, o/n). Finally, and as expected, this is reversed in DIPP1/5-IP$_7$ in the presence of NaF but fluoride has no effect on the complex with the product (C), further pointing to an enhancement of substrate processing into product as the major effect induced by Mg^{2+}.

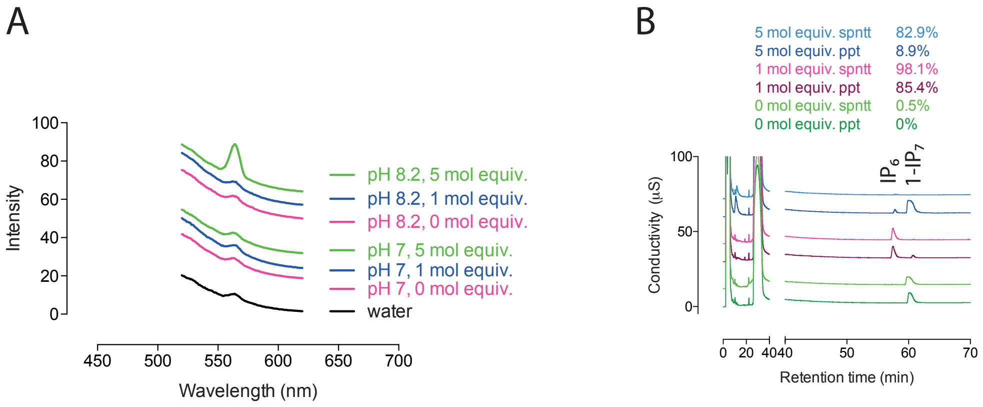

Fig. S7. Precipitation of IP_6 and 1-IP_7 at high Mg^{2+}.

(A) IP_6 (200 μM) was mixed with 0, 1 or 5 mol equivalent of Mg^{2+} in assay buffer (see above) at pH 7 and 8.2. Light scattering was determined in a spectrofluorimeter. Water scatters incident 280 nm excitation light. This passes the emission monochromator, as second-order diffraction. Only at pH 8.2 and 5 mol equivalent of Mg^{2+} is scattering increased above the 'intrinsic' Rayleigh scattering of pure water. These observations are consistent with the 'loss' of catalytic activity, observed for all three substrates under this condition, arising due to precipitation of substrate. (B) 1-IP_7 substrate (200 μM) was incubated with wt-DIPP1 at 0, 1 or 5 mol equivalent of Mg^{2+} at 8.2 for 15 min. The products were centrifuged at 24,000 × *g* for 15 minutes and the supernatant carefully removed. Half of the supernatant was added back to the original tube to resuspend any precipitate. The original supernatant and resuspended precipitates were diluted with NaF-EDTA to resolubilize precipitate and analyzed by ion chromatography with suppressed ion-conductivity detection. The proportion of reactant recovered as IP_6 product is given as % for each pair of samples, supernatant (spntt) and precipitate (ppt). At 0 mol equivalent of Mg^{2+}, no product was formed in either of the paired samples. At 1 mol equivalent, substrate conversion was complete in the paired samples (that is, no or very limited substrate precipitation was observed), while at 5 mol equivalent of Mg^{2+}, virtually all the substrate was precipitated leaving little substrate available to enzyme. Even so, the % of conversion of soluble substrate to product is similar to that with 1 mol equivalent Mg^{2+}.

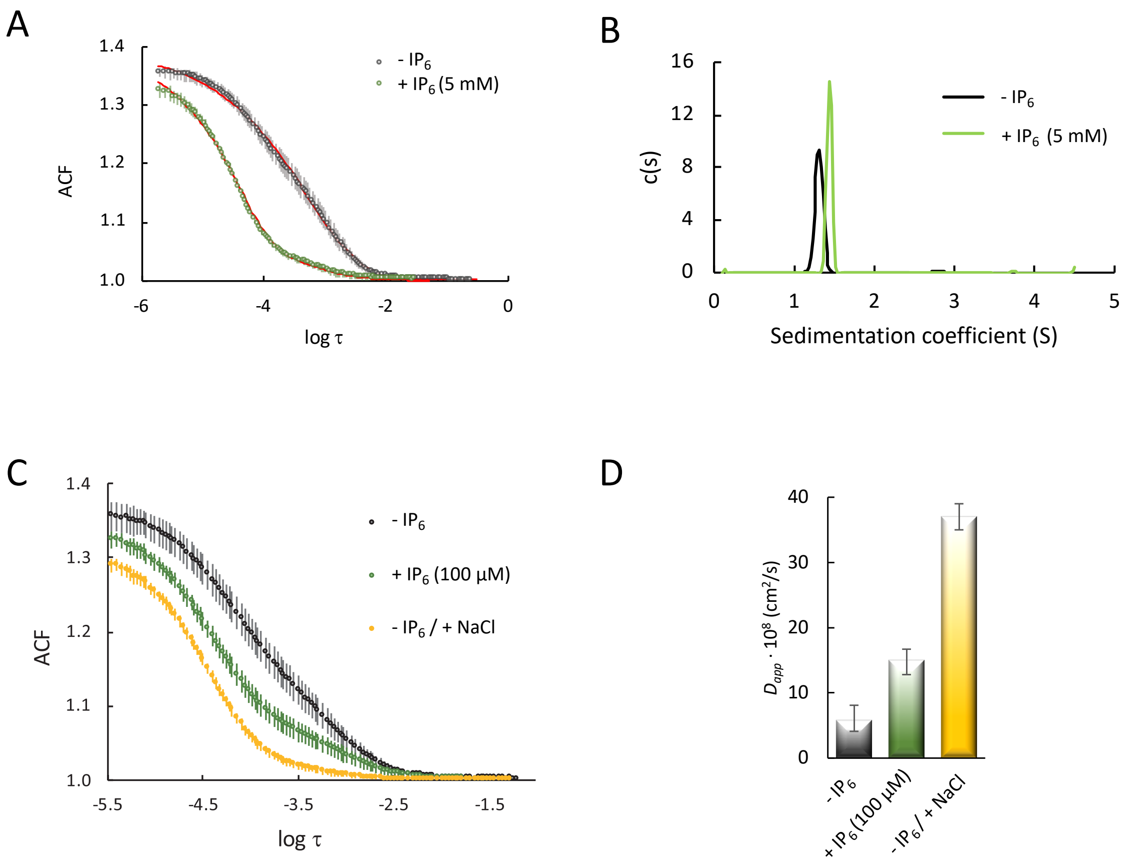

Fig. S8. Effect of IP_6 and salt on the hydrodynamic behavior of *Hs*DIPP1.
(A) DLS autocorrelation curves of wt-DIPP1 without and with 5 mM IP_6. Solid lines correspond to the fit of the models indicated in Methods. (B) Sedimentation profiles of wt-DIPP1, in presence of 150 mM NaCl, in absence (black) and presence (green) of the product IP_6 (5 mM). Sedimentation coefficient values are 1.3 S and 1.4 S, respectively. (C) DLS autocorrelation curves of wt-DIPP1 without and with 100 µM IP_6 or 150 mM NaCl. (D) D_{app} values obtained by fitting the one species model to the data in (C) as explained in Methods. DIPP1 concentrations were 100 µM (A), 155 µM (B) and 50 µM (C). All experiments performed in 20 mM Tris/HCl pH 7.5, 0.15 mM DTT, including 150 mM NaCl in (B) and in the yellow representations in (C and D).

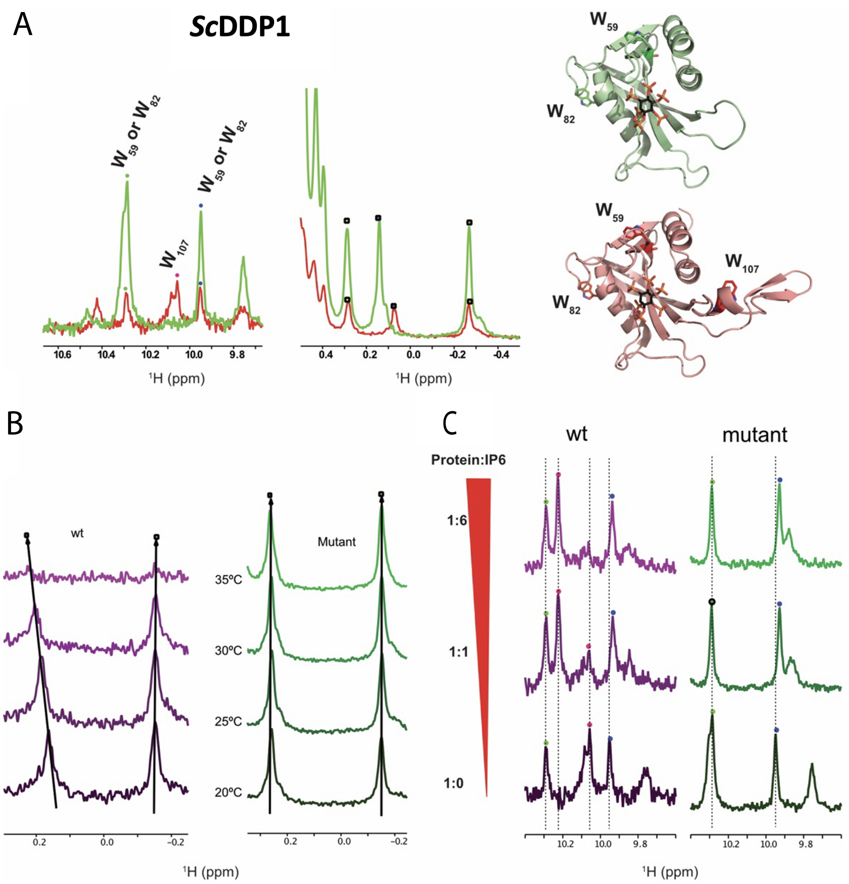

Fig. S9. ^{1}H NMR spectra of wt-*Sc*DDP1 *vs.* Δnose-*Sc*DDP1: effects of IP_6 and temperature.

(A) Superposition of the ^{1}H NMR spectra for wt-*Sc*DDP1 (red) and Δnose-*Sc*DDP1 (green) respectively, focusing on the upper-most and lower-most field resonances. Both spectra were recorded under the same experimental conditions. The signals corresponding to the Hε1 of the three (wt) and two (Δnose) *Sc*DDP1 tryptophan residues were tentatively assigned by sharpness and position; their location in the protein structures is shown in the figure on the right. The selected methyl signals likely correspond to the *Sc*DDP1 core and show shifts in the Δnose-*Sc*DDP1 constructs. (B) One of the methyl resonances shown in panel (A) exhibits temperature-dependent chemical shifts in the wt-, reflecting its participation in a chemical equilibrium (perhaps oligomerization) that is abolished in the mutant, and therefore depends directly on the nose region. (C) Effect of IP_6 titrations on the regions shown in panel (A). The signals of the two core tryptophans hardly change upon ligand addition, consistent with their long distance to the binding site. In contrast, Trp107 in the nose suffers a larger change and exhibits two resonances (bound and free) at sub-stoichiometric points. This tryptophan is located at a similar distance to the ligand binding site as the core ones; therefore, the changes are unlikely to be due to direct ligand contacts. Instead an indirect effect, like disruption of the oligomerization, seems a more plausible explanation for the spectroscopic behavior of Trp107.

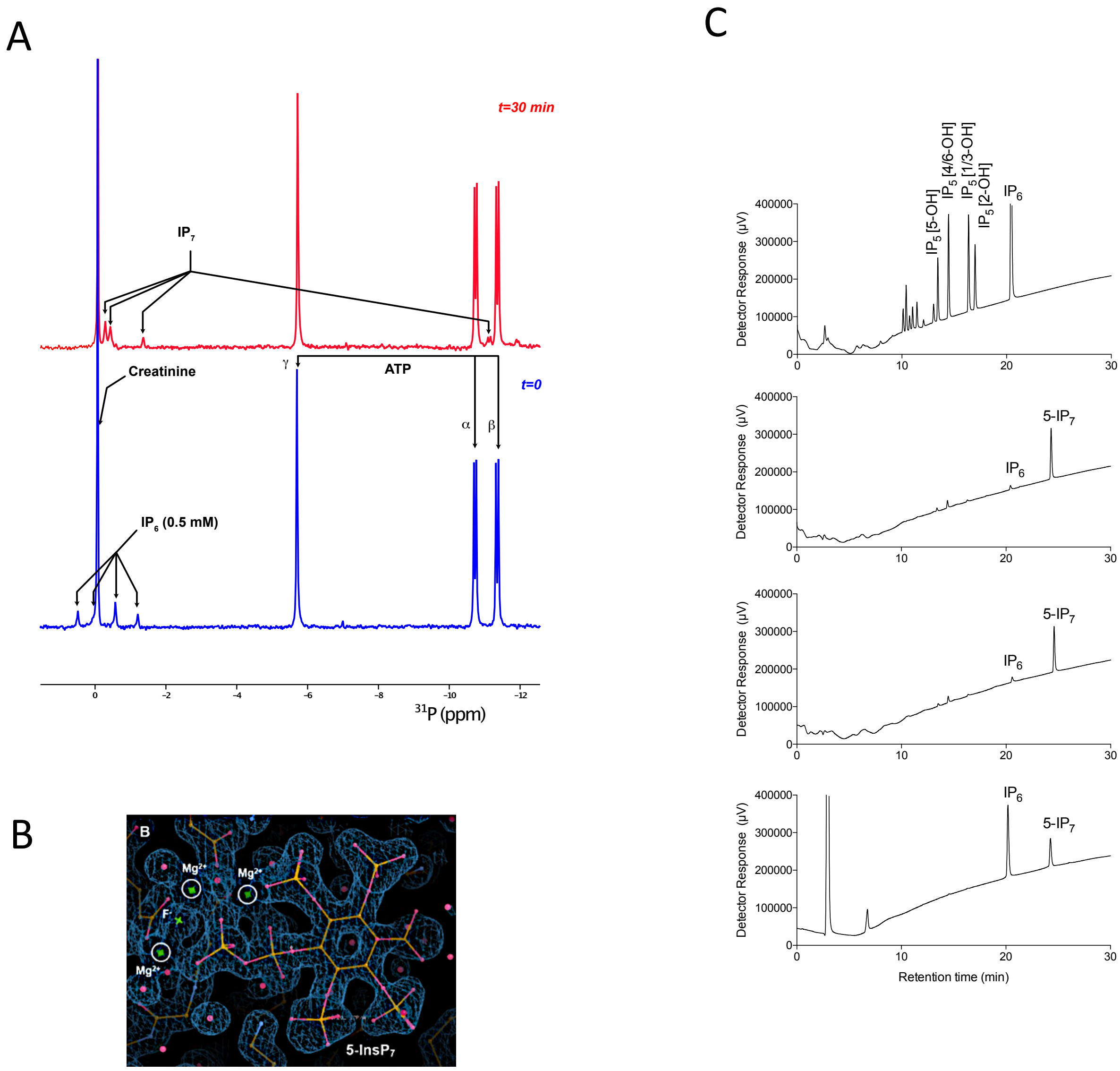

Fig. S10. Analysis of biosynthesized 5-IP$_7$.

(A) ^{31}P NMR spectra of IP$_6$ (blue) and biosynthesized 5-IP$_7$. The reaction catalyzed by *Hs*IP6K2 to synthesize 5-IP$_7$ from IP$_6$ and ATP reveals a quantitative substrate conversion into products over a time of 30 min. (B) Electron density map around 5-IP$_7$, in DIPP1/5-IP$_7$ co-crystals, contoured at 1 σ. (C) HPLC experiments showed a 75% yield in the biosynthesis reaction of 5-IP$_7$. Top trace: a hydrolyzate of IP$_6$ with (in reverse order) peaks of IP$_6$, IP$_5$ [2-OH], IP$_5$ [1/3-OH], IP$_5$ [4/6-OH], IP$_5$ [5-OH], then a collection of IP$_4$ isomers. Upper and lower middle trace (repetition): biosynthesized 5-IP$_7$ products showing (in reverse order) 5, 5-IP$_7$; 4, IP$_6$; 3, IP$_5$ [1/3-OH]; 2, IP$_5$ [4/6-OH]; 1 either IP$_5$ [5-OH] or I(1/3,4,5,6)P$_4$. Bottom trace: products of our assays of ITPK1 *vs.* IP$_6$ showing (in reverse order) 5-IP$_7$ and IP$_6$.
