# Supplementary material for: The DIPP1 family binds IP8 in catalytically-productive twist-boat and chair conformations and associates in a ligand-dependent manner
Source: Int J Biol Macromol. Author manuscript; Available in PMC 2026 Aug 1. (PMC7619153; doi:10.1016/j.ijbiomac.2026.152715)
Supplement: Supplementary Legends [file EMS214218-supplement-Supplementary_Legends.docx]

Supplementary videos 1, 2 and 3.

The three molecular dynamics simulations performed with wild-type DIPP1 and its natural substrate IP_8_, with 100 μs of production each. IP_8_ is shown as magenta sticks, and DIPP1 as white cartoons, with selected residues shown as white and cyan sticks. Oxygen and nitrogen atoms are shown in red and blue, respectively. Mg²⁺ ions are shown as green spheres.
